# Associations between genomic ancestry, genome size and capitula morphology in the invasive meadow knapweed hybrid complex (*Centaurea × moncktonii*) in eastern North America

**DOI:** 10.1093/aobpla/plz055

**Published:** 2019-08-23

**Authors:** Susanne Lachmuth, Jane Molofsky, Lindsey Milbrath, Jan Suda, Stephen R Keller

**Affiliations:** 1 University of Vermont, Department of Plant Biology, Burlington, VT, USA; 2 Martin Luther University Halle Wittenberg, Institute of Biology, Geobotany & Botanical Garden, Halle (Saale), Germany; 3 German Centre for Integrative Biodiversity Research (iDiv) Halle-Jena-Leipzig, Leipzig, Germany; 4 United Sates Department of Agriculture, Agricultural Research Service (USDA-ARS), Ithaca, NY, USA; 5 Charles University Prague, Department of Botany, Prague, Czech Republic; 6 Czech Academy of Sciences, Institute of Botany, Průhonice, Czech Republic

**Keywords:** Asteraceae, black knapweed, brown knapweed, *Centaurea jacea*, *Centaurea nigra*, genomic admixture, genotyping by sequencing, introgression, single nucleotide polymorphisms

## Abstract

Plant invasions are prime opportunities for studying hybridization and the nature of species boundaries, but hybrids also complicate the taxonomic treatment and management of introduced taxa. In this study, we use population genomics to estimate the extent of genomic admixture and test for its association with morphology and genome size in a hybrid complex of knapweeds invasive to North America: meadow knapweed (*Centaurea* × *moncktonii*) and its parental species (*C. jacea* and *C. nigra*). We sampled 20 populations from New York and Vermont, USA, and used genotyping by sequencing to identify single nucleotide polymorphisms in order to estimate genome-wide ancestry and classify individuals into hybrid genotype classes. We then tested for association between degree of genomic introgression and variation in a subset of traits diagnostic for the parental taxa, namely capitula morphology and monoploid genome size. Genomic clustering revealed two clearly defined lineages, as well as many admixed individuals forming a continuous gradation of introgression. Individual assignments to hybrid genotype classes revealed many advanced generation intercrosses and backcrosses, suggesting introgression has been extensive and unimpeded by strong reproductive barriers between taxa. Variation in capitula traits between the two unadmixed, presumed parental, lineages exhibited continuous, and in some cases transgressive, segregation among introgressed hybrids. Genome size was also divergent between lineages, although advanced generation hybrids had smaller genomes relative to additive expectations. Our study demonstrates deep introgression between the porous genomes of a hybrid invasive species complex. In addition to strong associations among genomic ancestry, genome size and morphology, hybrids expressed more extreme phenotypic values for capitula traits and genome size, indicating transgressive segregation, as well as a bias towards smaller genomes, possibly due to genomic downsizing. Future studies will apply these results to experimentally test how introgression, transgressive segregation and genome size reduction interact to confer invasiveness.

## Teaser

Hybridization is thought to be a primary trigger underlying plant invasiveness, yet identifying hybrids and differentiating them from their parental species presents formidable challenges. Here, we present a multifaceted analysis of the invasive meadow knapweed complex that unravels the genetic make-up of hybrid populations in North America using DNA sequencing to infer the genomic ancestry of individuals, and associating ancestry with variation in floral traits and genome size. We find extensive genomic evidence of hybrids, with significant but incomplete correspondence with floral traits and genome size. Our results highlight the widespread yet often cryptic nature of hybridization in plant invasions.

## Introduction

The invasion of numerous exotic plant species has been preceded or accompanied by interspecific hybridization. Consequently, hybridization has been hypothesized to be a key evolutionary mechanism stimulating invasiveness (Ellstrand and Schierenbeck 2000; Schierenbeck and Ellstrand 2009; Hovick and Whitney 2014). Admixture between parental genomes may increase the fitness of hybrids through short-term hybrid vigour (F1 generation) and/or increased evolutionary potential in later recombinant generations (Lavergne and Molofsky 2007; Keller and Taylor 2010; Keller *et al.* 2014; Parepa *et al.* 2014; Li *et al.* 2018). However, many plant taxa capable of hybridization also show partial reproductive isolation as a result of genetic incompatibilities, meiotic irregularities or gene dosage effects due to differences in genome size or copy number (ploidy), leading to either F1 hybrid sterility or outbreeding depression in advanced recombinant generations (Lynch 1991; Harrison and Larson 2014; Goulet *et al.* 2017). Consequently, the ways in which hybridization may facilitate invasion depend on whether hybrid genotypes are restricted to early generations (or maintained through clonal propagation), or rather if hybrid genomes are porous to introgression through advanced generations of recombination (Bock *et al.* 2015). Despite widespread appreciation of the association between hybridization and invasion (Ellstrand and Schierenbeck 2000), few studies have actually tested whether hybridization during invasion reflects short-term vs. advanced generations of introgression (Dlugosch *et al.* 2015).

One potential constraint on hybridization and how it resolves is the difference in genome size between parental taxa. In plants, hybridization is often associated with genome size variation, either due to genome duplication (polyploidy) or smaller-scale gains and losses of chromosomes (aneuploidy) or chromosomal segments during homoploid hybridization. Population-level cytogenetic estimation of genome size has proven successful in resolving complex hybrid taxonomies, delimiting species boundaries and highlighting the origins of hybrids (Mahelka *et al.* 2005; Ekrt *et al.* 2010). Additionally, frequent gains or losses of large genomic regions may have fitness consequences (Hovick and Whitney 2014), and there is a growing body of literature that supports the hypothesis that small holoploid (1C) genome size promotes invasion success (Pandit *et al.* 2014; Suda *et al.* 2014; Schmidt *et al.* 2017). Given the malleability of genome size to evolve differences between parental lineages and their hybrids (Baack and Rieseberg 2007), associating ancestry and genome size in an actively hybridizing invasive species complex could aid in distinguishing between parental types and hybrids, and may also yield insight into the reported associations of invasiveness with hybridization and genome size (Hovick and Whitney 2014; Suda *et al.* 2014).

An additional consequence of the increased phenotypic variance that often accompanies hybridization is that it may be difficult or impossible to determine the ancestry status of hybrids and their parental types using morphological or cytological data alone. This can complicate the study and management of invasive species as a consequence of taxonomic confusion over invading taxa, which are often recognized based on visually diagnostic morphological traits. Recent developments in population genomics provide vast opportunity for investigations into the genomic consequences of hybridization, even in non-model organisms lacking a reference genome (Goulet *et al.* 2017). The increased resolution of large, genome-wide sequencing data sets can prove advantageous to deciphering the pattern of ancestry and introgression, especially in morphologically difficult species complexes (Vilà *et al.* 2000).

One such putatively hybrid species is meadow knapweed (*Centaurea* × *moncktonii*)—one of the invasive representatives of the extremely polymorphic *Centaurea* subg. *jacea* (Asteraceae), sometimes referred to as the *C. jacea/nigra* complex (e.g. Gardou 1972). Meadow knapweed is reported to be a fertile hybrid between *C. jacea* (brown knapweed) and *C. nigra* (black knapweed), with either brown or black knapweed as the female parent and the hybrid capable of freely backcrossing with either parental species (Roché and Coombs 2003). Hybrids of *C*. × *moncktonii* were likely introduced to North America directly, along with the parental species (Roché and Roché 1991), from the late 1800s onwards, but the occurrence of more recent post-introduction hybridization events is also possible. Based on morphological identification, pure parental forms do not appear to persist at least in western North American hybrid zones (Roché and Roché 1991). So far, invasive *C.* × *moncktonii* populations are reported to be tetraploid, although both tetraploids and diploids occur in Europe (Roché and Roché 1991). Despite their relevance as noxious weeds in the USA (Roché and Coombs 2003), members of the complex have only been studied in Europe where it is native and not in North America (Garcia-Jacas *et al.* 2000, 2006; Vanderhoeven *et al.* 2002), and no genetic studies have resolved the ancestry of parental and hybrid types within this complex on either continent. Furthermore, it is not clear whether North American populations represent a hybrid complex of multiple knapweed species or whether only *C.* × *moncktonii* persists as advanced generation hybrids following the original introduction. Consequently, the potential effects of genomic recombination and genome size variation on invasive spread remained entirely unexplored.

Here, we report on a study that combines a population genomic survey of single nucleotide polymorphisms (SNPs) with morphometric analyses on reproductive traits and assessments of genome size for samples of the *C. jacea/nigra* complex collected from 20 locations in the northeastern USA (New York and Vermont). Our goal is to integrate multiple sources of evidence to deconvolute this apparent hybrid swarm into its constituent sources of ancestry and to determine the level of introgression that exists among parental species and their hybrids. Unravelling the extent of genomic introgression and mixed ancestry present within hybrid zones of introduced species is prerequisite for addressing the broader goal of how admixture and introgression affect fitness and contribute to invasion success (Keller and Taylor 2008; Dlugosch *et al.* 2015). Our results reveal an abundance of advanced generation hybrids (F2 and backcrosses) and the persistence of a much smaller number of individuals with unadmixed genomic ancestry. The genomic differences among ancestry groups are corroborated by strong associations between ancestry, the capitula traits measured and genome size. We discuss how combining multiple sources of evidence (genomics, cytometry, morphology) provide distinct advantages in deciphering the composition of plant hybrid zones, and can help unravel the complex systems of admixture that often arise during biological invasion.

## Materials and Methods

### Study species

*Centaurea jacea* and *C. nigra* (Asteraceae) are short-lived, perennial knapweeds native throughout Europe and introduced to North America. The taxonomic classification of the *C. jacea*/*nigra* complex is highly controversial; treatments range from a single polymorphic species (Briquet and Cavillier 1931) to several dozen species (van Soest 1947; Dostál 1976). In Northwestern Europe, the complex is extremely polymorphic presumably due to hybridization and polyploidy (Hardy *et al.* 2000), but genetic or cytological studies of this complex are lacking in North America. For this study, we followed the *Flora of North America* (Keil and Ochsmann 2006) by recognizing *C. jacea* and *C. nigra* as two different species. Referring to intermediates between *C. jacea* and *C. nigra*, including spontaneous interspecific hybrids, the *Flora of North America* follows Wagenitz (1987, in Orfeo Crosa and Bancheva 2006) in recognizing the nothospecies *C*. × *moncktonii*. According to Susanna and Roché (2011), *C. pratensis* and *C. debeauxii* are among previous names widely used for the hybrid between *C. nigra* and *C. jacea*, and the *Flora of North America* uses the epithet *pratensis* at the subspecific level, i.e. treating hybrids as nothosubspecies *C. jacea* subsp. × *pratensis* (Susanna and Roché 2011).

In their native Eurasian ranges, *C. jacea* has the widest native range with a rather continuous distribution spanning from Morocco to Norway and from Spain to Eastern Russia. The longitudinal distribution of *C. nigra* is more restricted, with easternmost records from Poland and Norway (Botanic Garden and Botanical Museum of Berlin-Dahlem 2006). The occurrence of *C. jacea* × *C. nigra* hybrids has been reported from several European countries, including the British Isles (Marsden-Jones and Turril 1954), Spain (Dostál 1976), Belgium (Vanderhoeven *et al.* 2002) and France (Orfeo Crosa and Bancheva 2006).

In North America, occurrences of *C. jacea* and *C. nigra* were reported before records of *C*. × *moncktonii* occurred, while the increasing spread of *C*. × *moncktonii* went along with an apparent decline of the parental taxa since the 1950s (Roché and Roché 1991). The species were thought to have been introduced unintentionally via ship ballast as well as intentionally as a hay or forage crop and as a pollen source for honeybees (Roché and Coombs 2003). The first reports of *C. jacea* and *C. nigra* in North America are both from the Pacific Northwest. *Centaurea jacea* was found at Vancouver Island, 1887 and *C. nigra* in Pullman, WA, in 1895 (Roché and Roché 1991). The earliest records of hybrid meadow knapweed are from Oregon near Eugene, Lane County in 1918 (Roché and Roché 1991). Before 1960, *C.* × *moncktonii* was additionally reported from British Columbia, Washington and Montana according to (Roché and Roché 1991), who were able to show a substantial increase of the hybrid in these states by the time of their 1991 survey and added first records in Idaho. In recent decades, meadow knapweed has rapidly expanded its range and now occurs in ~25 of the US and four Canadian provinces (Miller and Lucero 2014), ranging from the Atlantic to the Pacific and South to North Carolina (USDA, NRCS 2013 in Miller and Lucero 2014). It has greatly increased in abundance in western coastal states (Miller and Lucero 2014) and is common in parts of New York State and New England (Eckel 2012). Other Eurasian *Centaurea* species introduced to North America are not known to be reproductively compatible with the *C. jacea* s.l. species complex, with the exception of *C. nigrescens* which appears closely related and suspected to hybridize (Keil and Ochsmann 2006), and also rare observations of a sterile hybrid between *C.* × *moncktonii* and yellow starthistle, *C. solstitialis* (Susanna and Roché 2011).

*Centaurea jacea*, *C. nigra* and *C.* × *moncktonii* hybrids share various morphological and life history traits. All taxa are short-lived perennials flowering typically from July through October. They are insect-pollinated and self-incompatible (Hardy *et al.* 2001), reproducing primarily by seed. Whereas the taxa are hardly distinguishable based on vegetative traits (Keil and Ochsmann 2006), various capitula traits have proven diagnostic for members of the species complex (Hardy *et al.* 2000; Vanderhoeven *et al.* 2002; Keil and Ochsmann 2006) and are summarized for the parental species in **Supporting Information—Table S1.3**. North American meadow knapweed hybrid populations are quite variable morphologically and approach either parental species in appearance. In North America, *C. nigra* and *C. jacea* are commonly found in fields, pastures, roadsides, disturbed areas, clearings and waste places, although observations of *C. nigra* encompass a lower altitudinal range (0–300 m) compared to *C. jacea* (50–1300 m) (Keil and Ochsmann 2006). The hybrid *C.* × *moncktonii* prefers moister habitats than its parental species, such as meadows, irrigated pastures, riparian zones and moist forest openings (Roché and Roché 1991; Miller and Lucero 2014). The presence of the hybrid is undesirable because of its persistence in native plant communities and it also reduces the quality of forage produced in pastures and grass hayfields (Roché and Coombs 2003).

Hardy (2000) reported that *C. jacea* and *C. nigra* (studied collectively as *C. jacea* s.l. in that paper) occur as two distinct cytotypes in Europe: a diploid (2*n* = 22) and a tetraploid (2*n* = 44) (Hardy *et al.* 2000). Hybridization within cytotypes is relatively common, but hybridization between different cytotypes is rare (Hardy *et al.* 2000, 2001; Koutecky 2008). Therefore, hybridization appears to be homoploid (i.e. 2*n* × 2*n* or 4*n* × 4*n*) and produces offspring with the same ploidy level as their parents. Segregation of allozyme loci in controlled crosses between tetraploid genotypes suggests inheritance patterns vary among loci from strict tetrasomic to intermediate between tetrasomic and disomic (Hardy *et al.* 2001; Stift *et al.* 2008). Thus, it is likely the *4n* cytotype is in the process of diploidization of the homeologous parental chromosomes. The tetraploids also appear more phenotypically variable and have a wider ecological amplitude and geographical distribution than diploids (Gardou 1972).

### Sampling

**Figure 1.**
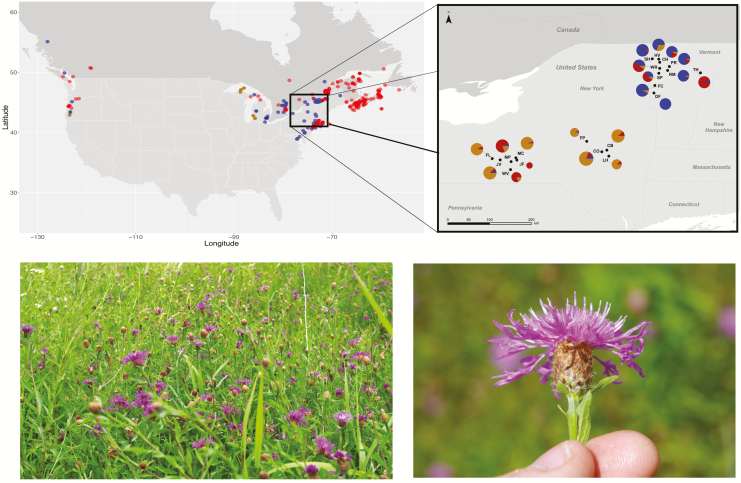
Range map showing North American occurrences of *C. jacea* (blue), *C. nigra* (red) and *C. × moncktonii* (orange) in the GBIF biodiversity database (gbif.org). Taxonomy was based on the determination accompanying each observation in GBIF. Inset map shows sampling locations from the current study of the *C. jacea/nigra* species complex in New York State and Vermont (USA). The 20 populations (see Table 1) are represented by pie graphs showing their assignment probabilities to three genetic clusters as identified by the *K* = 3 analysis in Admixture (Alexander *et al.* 2009). The size of the pie graphs is proportional to the number of samples per population. Pictures show a representative field population and close-up of a capitulum. Image credits: Jeromy Biazzo.

In 2015, 20 populations (see Fig. 1; Table 1) of the *C. jacea*/*nigra* complex were sampled from each of 10 locations in New York and Vermont. For each population, a whole young leaf (100 mg) and 1–2 mature capitula were collected from each of a minimum of 20 individuals. Leaf samples were transported back to the lab, and stored frozen at −80 °C until DNA extraction. Capitula were stored dried in envelopes in the lab until morphological analysis.

**Table 1.** Sampling locations of the *C. jacea/nigra* species complex in New York State and Vermont, USA. N-GBS, sample size for genomic analyses (after removal of samples with >70 % missing genotypes); N-GS, sample size genome size data (number of seed families); N-morpho, sample size morphometric data (number of individuals); Taxon, taxon to which the majority of the population was assigned in Admixture (Alexander *et al.* 2009) analysis for *K* = 2; Gen. size, mean holoploid genome size (1C value, pg).

Population ID	Locality	State	Latitude (°N)	Longitude (°W)	N-GBS	N-GS	N-morpho	Taxon	Gen. size (1C, pg)
CB	Central Bridge, Intersection of Route 7 and 30A	NY	42.71138	74.31467	17	6	17	Hybrid	1.931
CO	Cobleskill, County Route 1	NY	42.67663	74.42293	20	10	10	Hybrid	1.960
FL	Finger Lakes National Forest	NY	42.52917	76.77743	15	7	7	Hybrid	1.902
FP	Fort Plain, Brookman’s Corners Road	NY	42.90015	74.74652	8	5	5	Hybrid	1.895
JF	Dryden, Gee Hill Road	NY	42.49807	76.24740	4	2	2	*C*. cf. *nigra*	2.025
JV	Jacksonville, Route 96	NY	42.50268	76.60673	17	8	10	Hybrid	1.891
LH	Middleburgh, Lawton Hollow Road	NY	42.58310	74.27177	10	6	9	Hybrid	1.905
MC	Mclean Bog	NY	42.54553	76.26913	16	6	10	Hybrid	1.926
MP	Ithaca, Mt. Pleasant	NY	42.46377	76.37228	17	8	6	Hybrid	1.986
WV	Willseyville, Willseyville Road	NY	42.29273	76.38383	10	6	5	*C*. cf. *nigra*	1.952
CH	Colchester, Chimney Hill	VT	44.59865	73.17804	13	6	3	*C*. cf. *jacea*	1.893
DF	Shoreham, Doolittle Farm	VT	43.93722	73.30215	13	4	3	*C*. cf. *jacea*	1.903
FC	Addison, Farr Cross Road	VT	44.10018	73.28369	16	7	3	*C*. cf. *jacea*	1.889
HV	Milton, Happy Valley Road	VT	44.66431	73.19869	15	8	5	*C*. cf. *jacea*	1.891
PR	Jericho, Packard Road	VT	44.50658	72.97207	14	7	5	*C*. cf. *jacea*	1.896
RM	Richmond, Park and Ride	VT	44.42362	73.00874	13	6	3	*C*. cf. *jacea*	1.889
SH	South Hero, Ferry Road	VT	44.67213	73.33817	15	7	3	*C*. cf. *jacea*	1.890
SP	South Burlington, Spear Street	VT	44.36105	73.19783	11	4	10	Hybrid	1.882
TH	Marshfield, Thistle Hill Road	VT	44.37105	72.30404	14	7	9	*C*. cf. *nigra*	1.955
WR	South Burlington, Williston Road	VT	44.46829	73.17772	15	7	8	Hybrid	1.885

Voucher specimens from each population were collected and have been submitted to the Liberty Hyde Bailey Hortorium at Cornell University (herbarium@cornell.edu) **[see****Supporting Information—Table S1.1****]**.

### SNP genotyping

We extracted total genomic DNA from leaf tissue using the DNeasy 96 Plant kit (Qiagen, Valencia, CA), quantified each sample fluorometrically using QUANT-IT (Invitrogen) and normalized concentrations to 10 ng μL^−1^. Normalized DNA was used to prepare 96-plex genotype-by-sequencing (GBS) libraries following the Elshire protocol (Elshire *et al.* 2011). We individually digested 100 ng of sample DNA with the methylation-sensitive restriction enzyme *ApeK1* (New England Bio Labs) for 16 h at 75 °C, followed by ligation with 1.2 ng barcoded adapter sequences (adapter sequences followed Elshire *et al.* 2011) for 1 h at 22 °C and then heat killed at 65 °C for 30 min. Following ligation, 5 μL of each barcoded sample were pooled together (96 samples total/library), cleaned using the Qiagen PCR cleanup kit and eluted into 30 μL EB buffer. We used 6 μL of this cleaned, pooled library in a 50-μL PCR reaction (1× NEB *Taq* Master Mix; 25 pmol each forward and reverse primers) for 18 cycles, following conditions reported in Elshire *et al.* (2011). The resulting PCR library was cleaned with a GeneJet NGS cleanup kit (Thermo Fisher) using the entire 50 μL and run on a Agilent 2100 Bioanalyzer to verify the expected size distribution and lack of contaminating adapted sequences. Libraries passing QC were then sequenced (one 96-plex library per lane) for 100 bp single-end reads on an Illumina HiSeq 1500 by the Vermont Integrative Genomics Resource (VIGR) at the University of Vermont. In total, we sequenced four 96-plex libraries, encompassing 380 samples.

We assessed the quality of the raw sequence reads using FASTQC v0.10.1 (http://hannonlab.cshl.edu/fastx_toolkit). We then used the genotyping pipeline GBS-SNP-CROP (Melo *et al.* 2016) to clean and demultiplex reads, align them to the reference, call SNPs and genotypes, and apply filters to retain high-confidence variants for downstream analysis. Briefly, this consisted of the following steps: We cleaned raw reads using Trimmomatic 0.36 (Bolger *et al.* 2014) to remove bases leading and trailing bases with *Q*-score < 30 and truncate reads when the average *Q*-score in a 4-bp window fell below 30, followed by demultiplexing based on individual barcodes. Since no reference genome is available for *C. jacea* s.l. we assembled a GBS-specific reduced-representation mock reference *de novo* using the Velvet assembler (Zerbino and Birney 2008). The assembly was based on sequences from five individuals (CB_18, LH_3, MC_10, CH_15 and LH_18) that comprised a large number of reads and possessed capitula traits representative of the morphological variation found across our sample. We optimized assembly parameters using the wrapper program VelvetOptimiser (https://github.com/tseemann/VelvetOptimiser), and searched a range of *k-*mer lengths between 19 and 31 (command line: VelvetOptimiser.pl -s 19 -e 31 -f ‘-short -fastq CB_18.R1.fastq LH_3.R1.fastq MC_10.R1.fastq CH_15.R1.fastq LH_18.R1.fastq’). The optimal settings had a hash length of 29, which produced an assembly of 854 446 contigs with an N50 of 100 bp, for a total assembled length of 94.2 Mb. While larger kmer values could have been searched and may have yielded longer contigs in some cases, larger kmer values are also computationally more demanding to search, and the highly fragmented nature of single-end GBS sequence ‘tags’ is not expected to assemble into large contiguous sequences (Andrews *et al.* 2016). We also explored *de novo* assembly of a mock reference by clustering sequences based on percent identity using the VSEARCH algorithm, which does not depend on kmer length. The average cluster length with VSEARCH (82 bp; range 32–123 bp) was very close but slightly smaller than obtained with Velvet, indicating that assembly of the GBS tags was not overly sensitive to assembly algorithm. We used the Velvet assembly as a reference sequence to map reads from all samples using the bwa-mem algorithm in BWA 0.7.12 with default parameters (Li 2013).

We used the SAMtools 1.1 (Li *et al.* 2009) mpileup to generate an alignment of potential SNPs, using only uniquely mapped reads and applying the –C50 flag to down-weight mapping quality in reads with excessive mismatches. The mpileup output was parsed, putative indels excluded and the resulting genotyping master matrix of all putative SNP positions across all individuals was used for SNP and genotype calling.

We applied a series of quality control filters to eliminate low-confidence SNP and genotype calls. To reduce PCR bias or sequencing and alignment artefacts, we filtered for: (i) minimum and maximum average depth per genotype of 3 and 200 reads, respectively, (ii) a minimum frequency of 70 % of individuals with genotype data to accept a SNP and (iii) a minimum of three individuals possessing the putative secondary allele at a minimum depth of three reads. We applied the additional constraints of: (iv) minimum depth of three reads required to call homozygotes when the secondary allele depth was 0, (v) minimum primary read depth of 3 required to call homozygotes when the secondary allele depth was 1 and (vi) minimum read depth of 3 required for *each* allele (primary and secondary) to call heterozygotes. To eliminate SNPs that were not strongly bi-allelic, we required (vii) a minimum of 90 % of non-primary allele reads to be the secondary allele. This filter was specifically applied to reduce multi-allelic SNPs and enrich for loci exhibiting disomic inheritance. Lastly, we (viii) eliminated SNPs in contigs that likely represent microbial contamination by running nucleotide searches of the mock reference against the NCBI nr database with BLASTn (Altschul *et al.* 1990) and excluded SNPs located in contigs that matched non-plant genomes.

After filtering, the genomic data set consisted of 25 999 polymorphic SNPs for 380 samples. To reduce the impact of missingness on downstream analyses, we imputed missing genotypes using the LD-kNNi algorithm as implemented in LinkImputeR (Money *et al.* 2017). We performed test runs to optimize imputation accuracy using read depth thresholds between 3 and 12 and missingness (individual and site) thresholds between 0.4 and 0.7 in increments of 0.1. For these test runs, we masked 5000 genotypes with a site read depth ≥20 assumed to represent accurately called genotypes, and estimated imputation accuracy as the proportion of masked genotypes that were imputed correctly. For all test runs, we filtered for a maximum combined read depth of 100 and a minor allele frequency of 0.05. Based on the test runs, we then imputed genotypes that had a combined read depth >4 after first excluding SNPs and then individuals with >70 % missing values. These settings lead to a high imputation accuracy of 0.9 in test runs, while retaining a reasonable number of individuals (273) and SNPs (17 035) in the data set. We then used bcftools (http://samtools.github.io/bcftools/) to convert genotypes with an imputed probability <0.9 to missing, followed by removing SNPs with >50 % missing values. Lastly, to eliminate loci departing from disomic, Mendelian segregation due to formation of tetravalents or gene paralogy, we filtered SNPs with significant heterozygote excess (*P* < 5 e^−6^ based on Bonferroni correction). Positions with heterozygote deficit were not removed since this is an expected phenomenon in a hybrid zone due to a Wahlund effect. The final data set contained SNP genotype data for 273 individuals at 10 348 polymorphic loci.

### Population structure, genomic admixture and hybrid classification

To identify signals of genetic ancestry and assess the degree of admixture in our sample, we performed maximum likelihood genotypic clustering using the program Admixture v1.3 (Alexander *et al.* 2009) to probabilistically assign individuals ancestry in one or more *K* genetic clusters. We tested different models of *K* from 2 to 20, and determined values of *K* most consistent with the data based on minimizing the 5-fold cross-validation error. To complement the Admixture analyses with a model-free clustering approach that does not make any genetic assumptions (i.e. Hardy–Weinberg, linkage equilibrium, disomic or tetrasomic inheritance) (Ortego *et al.* 2017), we performed a principal component analysis (PCA) using the R package Adegenet (Jombart and Ahmed 2011).

Assignments of genotypic ancestry can provide evidence for admixture in a sample, but cannot distinguish between different hybrid genotype classes (e.g. F1, F2, backcrosses, etc.). We used the program NewHybrids (Anderson and Thompson 2002) to explore whether admixed individuals were first generation (F1) hybrids or instead represented more complex scenarios of introgression arising from advanced generation inter-mating (F2+) or backcrossing. We used the R package *hybriddetective* (Wringe *et al.* 2017b) to assemble and test diagnostic marker panels and create simulated data sets of parental taxa and four hybrid genotype classes. To this end, we assigned 27 samples to each putative parental species based on ancestry (*Q*) values > 0.9 in the *K* = 2 Admixture run (Vähä and Primmer 2006). Individuals in these clusters morphologically resembled *C*. *jacea* and *C*. *nigra* to a large degree, and were thus labelled *ad hoc* as *C.* cf*. jacea* and *C.* cf*. nigra*, respectively (see also next section). We emphasize the use of ‘cf.’ here, from the Latin ‘confer’ or ‘compare to’, as an indication of the resemblance of our sample to the above referenced taxa without asserting a formal taxonomic assignment. These data were further subdivided to use half the samples per parental taxon for marker selection and hybrid simulation, respectively.

NewHybrids assumes loci are independent (i.e. unlinked) and show strong allele frequency differences between parental taxa. To select an optimal subset of loci, we used the *getTopLoc* function to select diagnostic markers with low linkage disequilibrium within a parental taxon (assessed with PLINK; Purcell *et al.* 2007) and high *F*_S__T_ (assessed with *hierfstat*; Goudet 2005) between parental taxa. To account for choice of parental taxon when developing diagnostic marker panels, we performed marker panel selection separately for both the *C.* cf*. jacea* subsample and the *C.* cf*. nigra* subsample. In application, we found that these two diagnostic panels returned slightly different estimates for some hybrid classes (see Results), probably reflecting unknown differences in genomic structure and recombination in each lineage. Rather than combine the two sets of markers into a single panel, we instead report hybrid assignments from each panel separately so that sensitivity and agreement in the hybrid class assignments can be assessed. We analysed simulated data sets of the two parental taxa as well as F1, F2 and F_1_ backcrosses using panels of 200, 300, 400 and 500 diagnostic markers. We then analysed each of nine replicates of the simulated data sets with the R package *parallelnewhybrid* (Wringe *et al.* 2017a) in order to determine the accuracy, efficiency and overall power of classification with the different marker sets. We used Jefferey’s prior probabilities and ran the Markov chain for 300 000 iterations following a burn-in of 50 000 iterations. Based on simulation results, we chose a 500 marker panel to analyse our combined empirical and simulated genotypes with NewHybrids, with simulated samples of the parental taxa flagged as known. For these analyses, we allowed assignment to additional hybrid classes including two generations of backcrossing F1’s to the parental taxa **[see****Supporting Information—Table S1.2****]**. We note that hybrid assignment beyond the F1 cannot distinguish among the different classes of advanced generation hybrids (F2, F3, etc.), since these share the same global ancestry probabilities.

### Morphometric and genome size associations with inferred genomic ancestry

**Figure 2.**
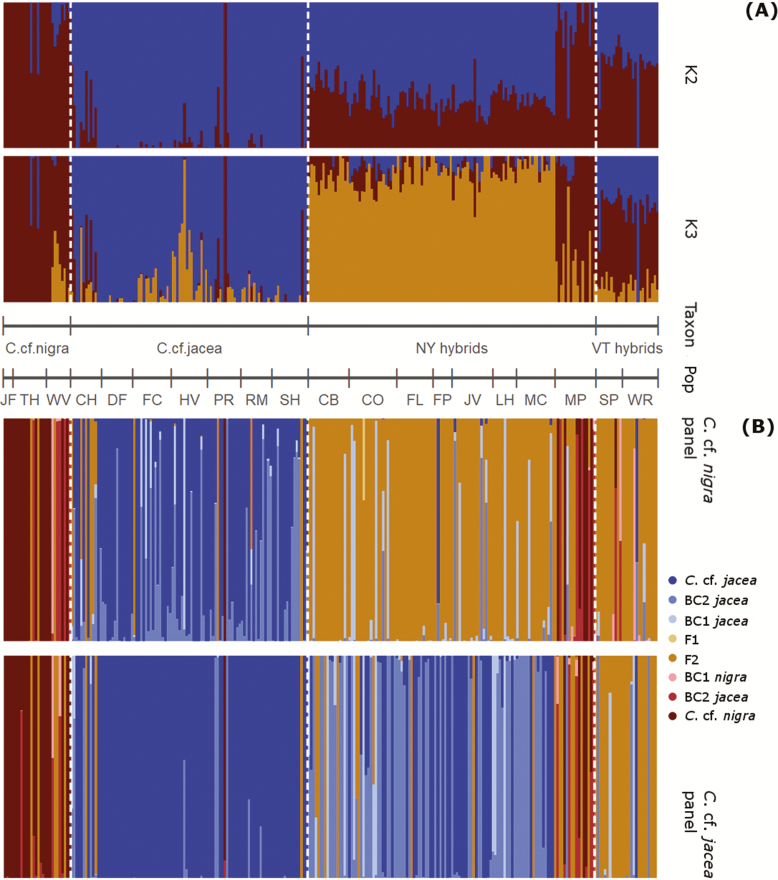
Assignment of 273 individuals of the *C. jacea/nigra* species complex sampled in New York State and Vermont to genetic clusters and hybrid classes. Population labels follow Table 1. (A) Individual probabilities of assignment to *K* = 2 and to the optimal *K* = 3 genetic clusters identified by Admixture based on 10 348 SNPs. (B) NewHybrids individual assignments to the presumed parental taxa and various hybrid classes. The classes were derived from the NewHybrids analysis using a set of 1000 selected SNP loci (i.e. marker panels) that showed highest global *F*_ST_ and no linkage disequilibrium among 27 *C*. cf. *nigra* (upper panel) and *C*. cf. *jacea* (lower panel) individuals.

Because the taxonomic status of North American populations of the *C. jacea*/*nigra* complex is unresolved, we assessed whether genetic clusters identified by Admixture were associated with differentiation in some of the morphological capitula traits previously used to characterize this species complex (Vanderhoeven *et al.* 2002). We measured a subset of individuals with high ancestry assignments (*Q* > 0.9) from the *K* = 2 Admixture model (*N* = 22 and 25 individuals per cluster) along with 86 individuals with mixed genomic ancestry randomly chosen from all sampled populations (Table 1; Fig. 2A). From each of these individuals, we created standardized digital images of 1–2 field-collected capitula and used *ImageJ* (Schneider *et al.* 2012) to measure capitula traits. We focused on a subset of traits reported in the *Flora of North America* that distinguish *C. jacea* and *C. nigra* (Keil and Ochsmann 2006) and/or that have proven diagnostic in previous morphometric analyses on European samples of this species complex (Hardy *et al.* 2000; Vanderhoeven *et al.* 2002) **[see****Supporting Information—Table S1.2****]**. Unlike Hardy (2000) and Vanderhoeven (2002), we chose to analyse trait ratios (e.g. width:length) when possible, since not all capitula were in exactly the same developmental stage. We statistically compared trait values of the two unadmixed genetic clusters using linear mixed models with a random effect of individual nested within population using the R package ‘*lme4*’ (Bates *et al.* 2015). Traits that showed significant differences between unadmixed genetic clusters **[see****Supporting Information—Fig. S1.1****]** were included in a morphometric PCA using all individuals and the R package ‘*stats*’ (R Core Team 2017). We then used linear mixed models with population as random effect to analyse the dependence of individuals’ mean axis scores for morphometric principal components 1 and 2 (response variables) on their axis scores for genetic principal components 1 and 2, as well as their estimated NewHybrids hybrid class.

As a further tool to help discriminate the ploidy level of our sample and to help decipher parental and hybrid taxon assignments, we measured genome size by flow cytometry. Genome size (1C values) reported in the literature for tetraploid *C. jacea* is generally ~2 pg, with reported variation among closely related species as well as within cytotypes of *C. jacea* (Bancheva and Greilhuber 2005; Dydak *et al.* 2009). Thus, correspondence between genetic ancestry and genome size provides additional confidence in parental and hybrid taxon assignments that may be less subjective and environmentally plastic compared to morphometric traits. We sampled fresh leaf tissue for genome size from three open-pollinated offspring per field-collected individual. Seeds were germinated, grown in a greenhouse at the University of Vermont, and leaf tissue sampled when plants were large rosettes. Tissue was kept cool but not frozen and shipped to the Laboratory of Flow Cytometry at the Institute of Botany, Czech Academy of Sciences. Genome size was determined following the protocol described in (Doležel *et al.* 2007), using *Solanum lycopersicum* ‘Stupicke polni rane’ (1C = 0.98 pg) as an internal reference standard loaded with each sample.

We analysed differences in mean genome size and morphometric traits as a function of genetic ancestry and hybrid class using linear mixed models, with population as a random effect. When the main effect of hybrid class was significant, we applied Tukey’s *post hoc* tests for pair-wise comparisons of hybrid classes using the R package ‘*multcomp*’ (Hothorn *et al.* 2008). All analyses performed in R used R version 3.4.1 (R Core Team 2017).

## Results

### Population structure, genomic admixture and hybrid classification

The composition of genetic ancestry in our sample estimated by Admixture showed two relatively pure clusters at *K* = 2. These clusters separated individuals visually resembling *C. nigra* and *C*. *jacea* based on a subset of diagnostic capitula traits (treated tentatively here as *C*. cf*. nigra* and *C*. cf*. jacea*; see also section ‘Morphometric trait variation among the identified genetic clusters and hybrid classes’) as well as a large number of individuals with admixed genetic ancestry (Fig. 2A, upper panel, red for *C*. cf*. nigra* and blue for *C*. cf*. jacea*). Geographic populations that were assigned by majority to one of the two unadmixed clusters usually also contained some individuals with mixed ancestry (i.e. maximum assignment probability < 0.9 in the *K* = 2 run).

At *K* = 3, the model best supported by cross-validation in Admixture, most admixed individuals formed a separate cluster (orange), suggesting the presence of a distinct hybrid lineage with intermediate ancestry between *C*. cf*. nigra* and *C*. cf*. jacea* (Fig. 2A, lower panel). Of the 102 individuals that showed a majority assignment to the third hybrid cluster, 99 were collected from seven populations in New York that had majority assignment to *C.* cf. *nigra* (NY hybrids), with one additional individual in NY population MP (Figs 1 and 2A) and two individuals from Vermont populations HV and SH. Two additional admixed populations were from Vermont (SP, WR; VT hybrids) harbouring 26 individuals whose ancestries included all three clusters, but with highest assignment probability for *C*. cf*. nigra* (Figs 1 and 2A lower panel; Table 1).

Inference of each individual’s hybrid genotype class by NewHybrids (Fig. 2B) largely reflected the ancestry scores from Admixture; however, the hybrid class assignments slightly differed depending on whether the marker panel was based on *C*. cf. *jacea* or *C*. cf*. nigra* (see also **Supporting Information—Fig. S1.2**), with the assignments of backcross hybrid classes showing a trend towards the parent taxon that served as the source of marker selection. In both cases, several individuals from populations containing a majority of *C*. cf*. nigra* or *C*. cf*. jacea* were classified as hybrids, congruent with the Admixture results. Most individuals assigned to the orange hybrid cluster in the Admixture analysis were classified as F2 (or higher generation) hybrids based on the *C*. cf*. nigra* panel, or as second (or higher) generation backcrosses towards *C*. cf*. jacea* based on the *C*. cf*. jacea* panel (Fig. 2B). By contrast, the VT hybrids assigned to all three clusters in the Admixture analysis were mostly classified as F2 (or higher) hybrids in both NewHybrids analyses. No F1 hybrids and few first generation backcrosses were identified, suggesting most hybrids in our sample represent advanced generations of introgression.

**Figure 3.**
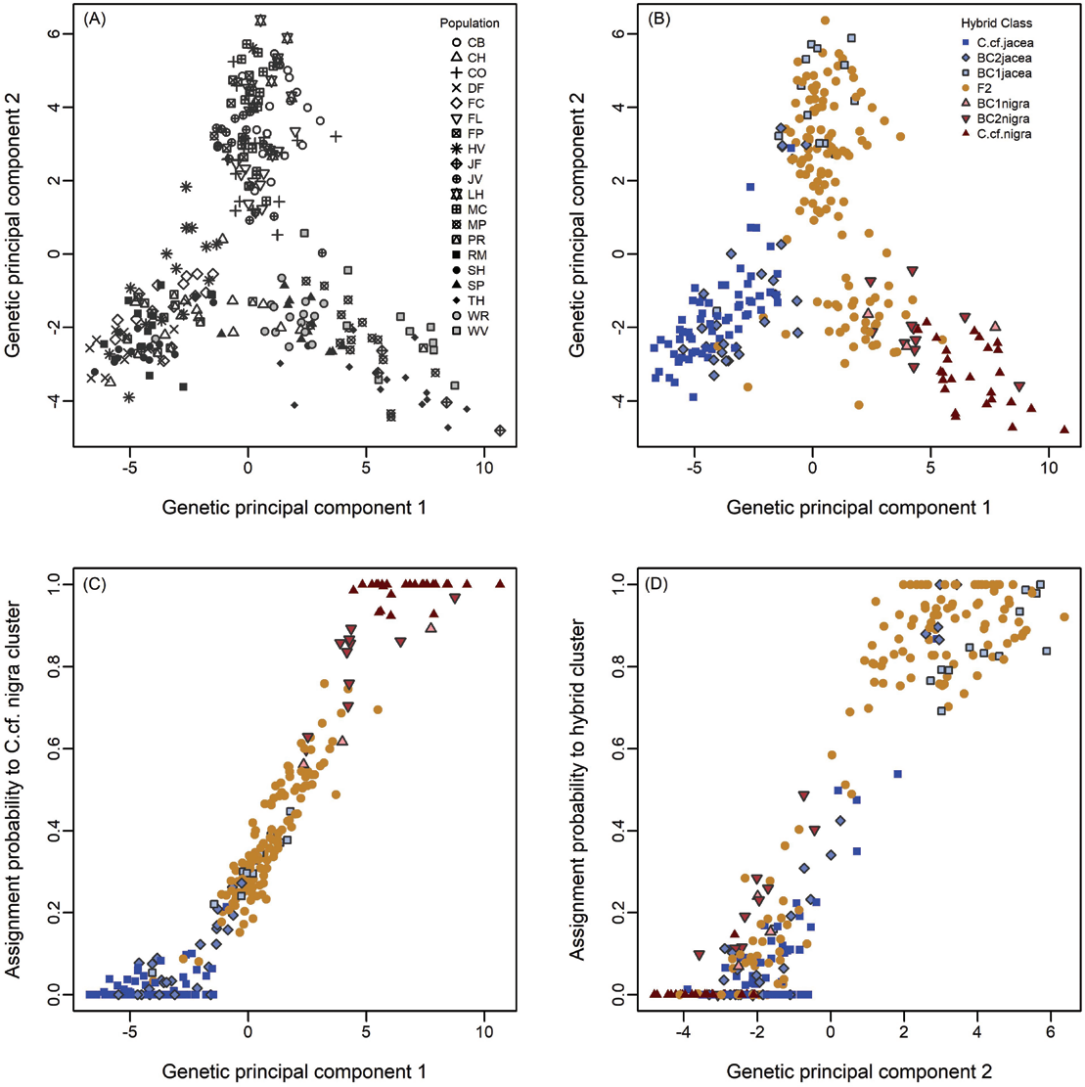
Genetic structure of 273 individuals of the *C. jacea/nigra* species complex sampled in New York State and Vermont based on 10 348 SNPs. (A, B) Principal component analysis with individuals labelled by source population and assigned hybrid genotype class. (C) Relationship between the Admixture assignment probability to the *C*. cf. *nigra* (red) cluster for *K* = 2 (Fig. 2A, upper panel) and the scores of genetic principal component 1. (D) Relationship between the Admixture assignment probability to the *C*. × *moncktonii* hybrid cluster (grey) for *K* = 3 (Fig. 2A, lower panel) and the scores of genetic principal component 2. Hybrid classes were derived from the NewHybrids analysis using the *C*. cf. *nigra* marker panel (Fig. 2B, upper panel).

The PCA based on all 10 348 SNPs identified three genomic clusters that closely corresponded with the Admixture and NewHybrids results based on the *C*. cf*. nigra* marker panel (Fig. 3A and B). The first two clusters consisted of parental *C*. cf*. jacea* and *C*. cf*. nigra* individuals, respectively, separated along principal component (PC) 1, while individuals with hybrid ancestry were intermediate (Fig. 3C). However, hybrid genotypes separated out into different positions along PC2—one group of hybrids composed primarily of F2 and BC1-jacea genotype classes showed high PC2 scores, corresponding to the orange Admixture cluster. The remaining hybrid individuals from genotype classes BC2-jacea, BC1-nigra, BC2-nigra had lower PC2 scores and clustered more closely with their respective parental taxa (Fig. 3A, B and D).

### Morphometric trait variation among the identified genetic clusters and hybrid classes

Due to better correspondence of hybrid genotype classes with genomic ancestry (Admixture, PCA) and capitula morphology (see below), we hereafter focus on the hybrid class assignments made using the *C*. cf*. nigra* marker panel. The corresponding results based on the *C*. cf*. jacea* marker panel are presented in **Supporting Information—Appendix S2**. Morphometric capitula traits showed distinct variation between but also high variation within the two putative parental taxa inferred based on unadmixed genomic ancestry **[see****Supporting Information—Table S1.3; Fig. S1.1****]**. However, three traits differed significantly (and in accordance with expectations) between the two presumed parental taxa: the percentage of pectinate bract rows was significantly higher in *C*. cf*. nigra* than *C*. cf*. jacea* (χ ^2^_(1)_ = 30.7, *P* < 0.001), whereas the ratio of bract appendage centre width to length (χ ^2^_(1)_ = 6.2, *P* < 0.05) and the relative bract appendage centre width (χ ^2^_(1)_ = 8.7, *P* < 0.01) were both significantly lower in *C*. cf*. nigra* than *C*. cf*. jacea*.

**Figure 4.**
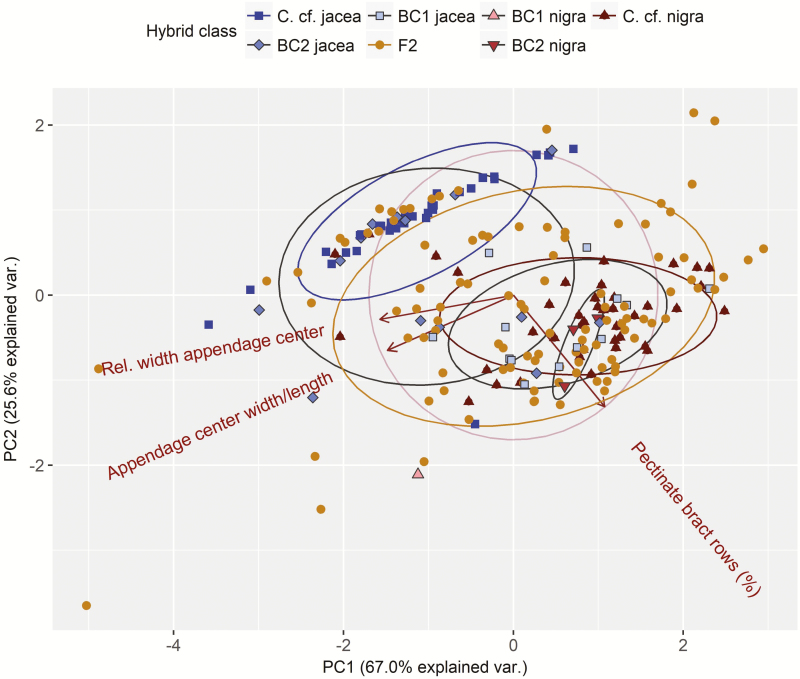
Principal component analysis based on three morphometric capitula traits **[see****Supporting Information—Table S1.3; Fig. S1.1****]** derived from diagnostic characteristics from previous morphometric analyses on European samples of the *C. jacea/nigra* complex (Hardy *et al.* 2000; Vanderhoeven *et al.* 2002). The traits were measured for 133 individuals using standardized digital images of one or two field-collected capitula per individual. Coloured labels correspond to hybrid classes derived from the NewHybrids analysis using the *C*. cf. *nigra* marker panel (Fig. 2B, upper panel).

**Figure 5.**
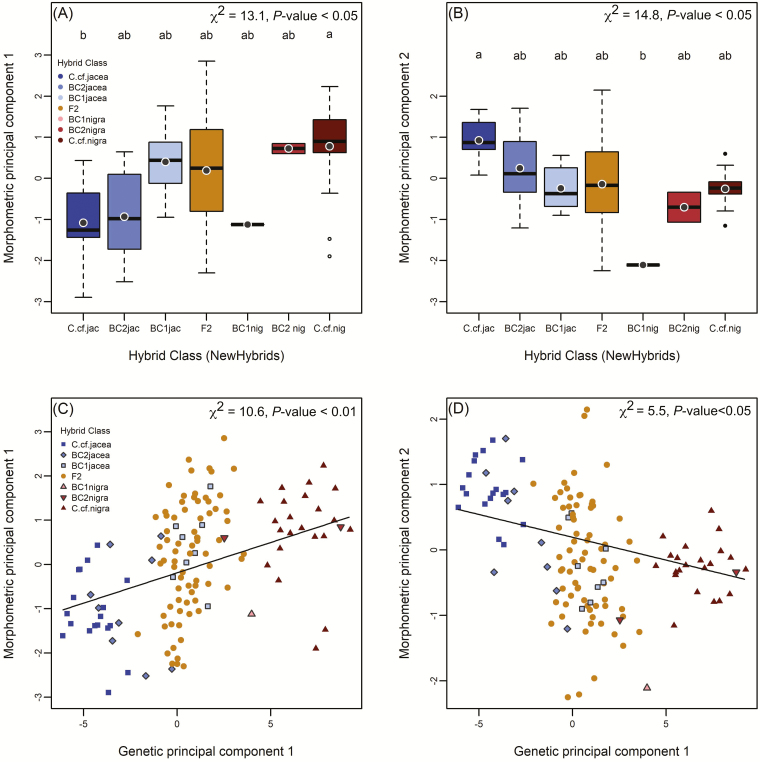
Associations between genetic ancestry and reproductive morphology. Mean individual scores for principal component 1 (A, C) and 2 (B, D) from the morphometric PCA (Fig. 4) predicted by NewHybrids hybrid class (A, B) or individual scores for genetic PC1 (C) and PC2 (D). Hybrid classes that do not share a lower-case letter differ significantly (*P* < 0.05) according to Tukey’s *post hoc* tests.

Consequently, the *C*. cf*. jacea* and *C*. cf*. nigra* individuals separated remarkably well in a PCA involving these three traits (Fig. 4). The hybrids, in particular the F2, exhibited transgressive segregation, exceeding parental trait values on the first two morphometric PC axes, which collectively explained 92 % of the trait variance. When comparing morphometric PC scores of the hybrid classes (Fig. 5A and B), we found significant differentiation between *C*. cf*. jacea* and *C*. cf*. nigra* individuals along morphometric PC1 (χ ^2^_(1)_ = 13.1, *P* < 0.05 for hybrid class main effect), whereas all classes of hybrids did not differ significantly from either of the presumed parental taxa in morphometric PC1 scores (Fig. 5A). For morphometric PC2, we did not find significant differences between the presumed parental taxa and hybrids except for significantly higher scores in *C*. cf*. jacea* than first generation backcrosses to *C*. cf*. nigra* (χ ^2^_(1)_ = 14.8, *P* < 0.05 for hybrid class main effect), but this difference should be interpreted with caution since the sample size in the latter hybrid class was quite small (Fig. 5B). Morphometric differentiation between hybrid classes was less pronounced when they were assigned based on the *C*. cf*. jacea* marker panel **[see****Supporting Information—Fig. S2.3A and B****]**.

The association between capitula morphology and genomic ancestry was further strengthened when regressing the morphometric PC scores against the genetic PC scores (Fig. 5C and D). Genomic ancestry (genetic PC1) significantly predicted variation in capitula morphology (morphometric PC1 χ ^2^_(1)_ = 10.6, *P* < 0.01; PC2 χ ^2^_(1)_ = 5.5, *P* < 0.05). These relationships were primarily driven by the clear morphological separation of genotypes classified as unadmixed parental taxa. By contrast, admixed hybrid individuals with intermediate values of genetic PC1 displayed highly variable capitula morphologies, but with a mean centred near the predicted value based on their ancestry (Fig. 5C and D). When categorizing hybrid classes based on the *C*. cf*. jacea* marker panel, the majority of this morphologically highly variable group of individuals was assigned to either *C*. cf*. jacea* or backcrosses to this parental taxon **[see****Supporting Information—Fig. S2.4A and C****]**. We did not find any significant relationships between genetic PC2 and either morphometric PC, which suggests that genomic differences that separate hybrid genotypes along genetic PC2 (Fig. 3B) are not reflected in capitula morphology **[see****Supporting Information—Figs S1.3A and B; S2.4B and D****]**.

### Genome size variation among the identified genetic clusters and hybrid classes

**Figure 6.**
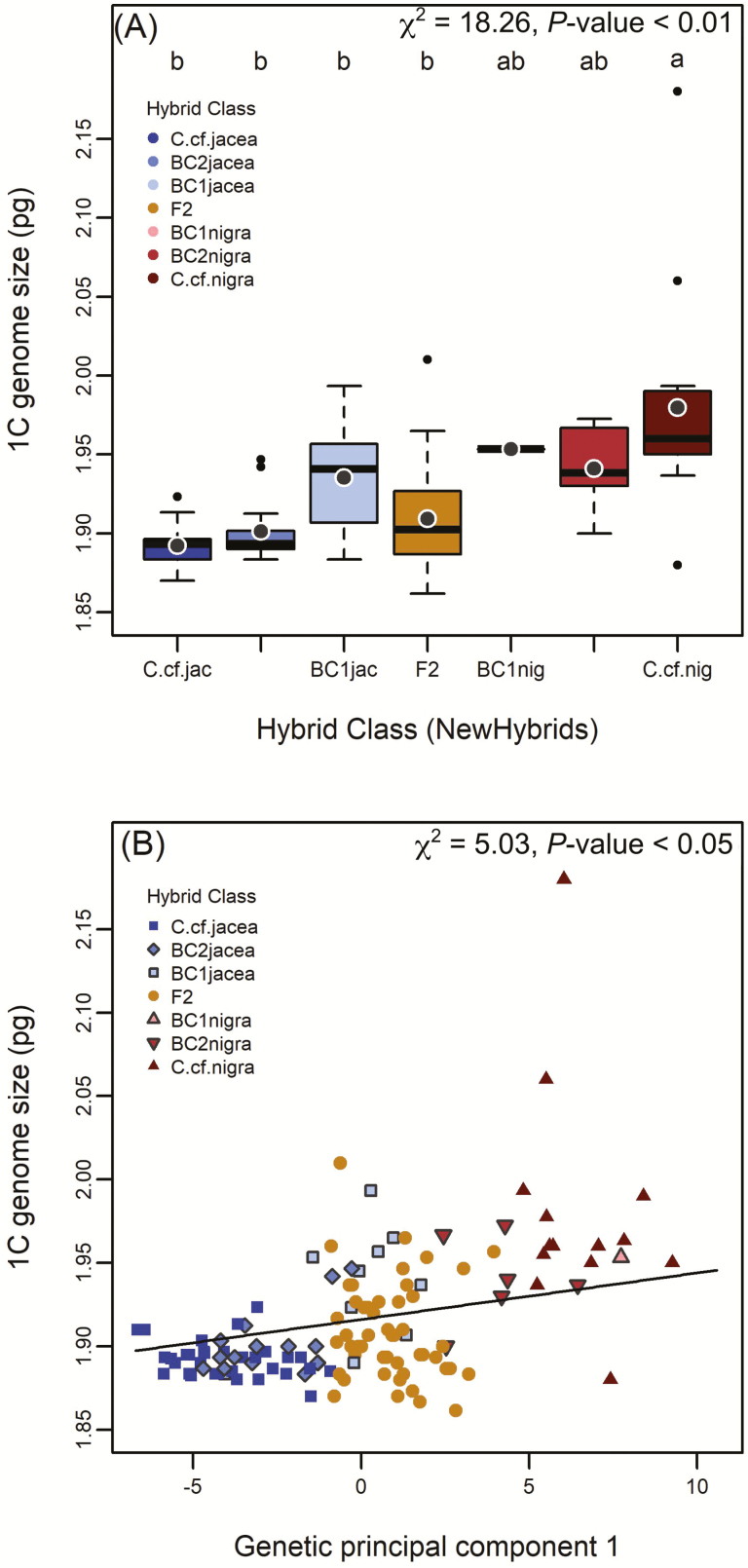
Associations between genetic ancestry and genome size. Mean offspring genome size (1C value) was predicted by NewHybrids hybrid class (A) and genetic PC1 (B). Each point represents an individual, and coloured labels correspond to hybrid classes derived from the NewHybrids analysis using the *C*. cf. *nigra* marker panel (Fig. 2B, upper panel).

Holoploid (1C) genome size of our samples ranged between 1.83–2.28 pg, consistent with all sampled individuals being tetraploid (Bancheva and Greilhuber 2005; Dydak *et al.* 2009). Sample variation within individuals was low (CV_*Solanum*_ = 3.19 %; CV_*Centaurea*_ = 2.75 %), indicating we achieved good precision for estimating individual genome size. Despite the uniformity in DNA ploidy, we found considerable genome size differences that reflected genomic ancestry. Among NewHybrids genotype classes, *C*. cf*. jacea* as well as all types of backcrosses towards this parental taxon and F2 hybrids had significantly smaller genomes than *C*. cf*. nigra* (Fig. 6A; χ ^2^_(1)_ = 18.3, *P* < 0.01 for hybrid class main effect). The intermediate genome sizes of backcrosses to *C*. cf*. nigra* did not differ significantly from any other hybrid class. Interestingly, advanced hybrid generations (F2 and second generation backcrosses) consistently tended towards smaller genome sizes as compared to first generation backcrosses. Similar trends for hybrid class assignments were observed based on the *C*. cf*. jacea* marker panel **[see****Supporting Information—Fig. S2.5A****]**.

In accordance with these results, we found a significant relationship between genetic PC1 and genome size (Fig. 6B; **see****Supporting Information—Fig. S2.5B**; χ ^2^_(1)_ = 5.03, *P* < 0.05). However, this effect was no longer significant when an extreme outlier (*C*. cf*. nigra* with a 1C value > 2.15) was excluded. Again, this relationship was mostly driven by genome size differences between the two parental taxa, whereas in the middle range of genetic PC1, genome size was highly variable. There were no significant relationships between genetic PC2 and genome size **[see****Supporting Information—Figs S1.4 and S2.5C****]**, or between morphometric PC scores and genome size **[see****Supporting Information—Figs S1.5 and S2.6****]**.

## Discussion

Interspecific hybridization is common in plant evolution and is an important source of novel trait architectures and adaptive variation (Rieseberg *et al.* 2003), yet most species are sufficiently reproductively isolated to prevent total dissolution of species boundaries. When weakly diverged taxa come into secondary contact, lack of strong reproductive barriers makes the parental genomes permeable to introgression, leading to extensive backcrossing and the formation of hybrid swarms (Harrison and Larson 2014). This process of genomic admixture and introgression may increase trait variance through the release of latent genetic variation, but also blurs the lines that keep species distinct. Such appears to be the case for the hybrid swarm formed between *C. jacea*, *C. nigra* and their hybrid, *C*. × *moncktonii*. This highly diverse, polymorphic species complex has been suggested to hybridize throughout its native European range, as well as in North America where it is introduced. However, much confusion exists about the separation of *C.* × *moncktonii* from its parental taxa based on morphology (Vonica and Cantor 2011), suggesting a population genomics approach could prove useful at shedding light on the composition of this hybrid swarm (Vanderhoeven *et al.* 2002).

Our results provide the first glimpse into the composition of genomic ancestry in the *Centaurea* × *moncktonii* hybrid species complex, focusing on introduced populations in the northeastern USA where hybrid meadow knapweed appears invasive. By associating genome-wide ancestry with variation in a subset of capitula traits and genome size, we obtained five key results: (i) both advanced generation hybrids as well as unadmixed parental types were found to occur in a geographically structured distribution across New York and Vermont; (ii) capitula morphology and genome size correspond significantly with genomic ancestry, reinforcing the phenotypic and genomic distinctiveness of the unadmixed lineages while also providing a detailed genetic view of the extent of their introgression; (iii) hybrid individuals showed great variation in floral traits and genome size, including transgressive segregation for capitula traits; (iv) many hybrids exhibited a *C.* cf*. nigra*-like capitula morphology although the majority of them had higher genomic ancestry from the *C.* cf*. jacea* parent; and (v) many advanced generation hybrids had relatively small genome sizes given their level of ancestry. We discuss these findings in detail below with an emphasis on how the combination of genomic, cytological and morphological data have provided our best view to date into the ancestry composition of this incredibly reticulate hybrid complex. The extensive introgression uncovered here offers opportunities for future research to shed light on how hybridization affects the ecological and evolutionary dynamics of invasive populations.

### Evidence of occurrence and geographic distribution of unadmixed parental-like taxa

In contrast to regions of the Pacific Northwest where *C*. × *moncktonii* hybrids have reportedly largely replaced populations of the parental taxa (Roché and Roché 1991), our results for the northeastern USA suggest that unadmixed genotypes still remain in the vicinity of an extensive array of advanced generation admixed genotypes (Figs 1 and 2A). Whether these taxa correspond precisely to the recognized taxonomy described for European *C*. *jacea* and *C*. *nigra* is uncertain at present, and will require the inclusion of native European samples in future analyses. Our preliminary classification of unadmixed individuals as *C*. cf*. jacea* and *C*. cf*. nigra* is based on congruent evidence from genomic, cytological and morphometric analyses, which all agree on the existence of genetically distinct lineages that correspond in most (but not all; see below) aspects of reproductive morphology to *C*. *jacea* and *C*. *nigra*. A third related taxon, *C. nigrescens* is also introduced to North America and is sometimes treated as part of the *C. jacea* s.l. complex, and *C*. × *moncktonii* is noted to sometimes combine features of *C. nigrescens*, notably the presence of involucres with distinct green phyllaries not fully covered by black appendages (Keil and Ochsmann 2006). Individuals possessing these traits were present but uncommon in our study area. However, another *C. nigrescens*-like trait was not clearly evident—the involucre being longer than wide (Keil and Ochsmann 2006). Thus, we cannot exclude the possibility that *C. nigrescens* has contributed some ancestry to this already complex hybrid swarm, but we think the dominant sources of ancestry in the hybrids we sampled originate from *C. jacea* and *C. nigra.* To our knowledge, no other reproductively compatible *Centaurea* species occur in our region.

The most common morphological characteristics used for taxonomic classifications of the genus *Centaurea* including hybrids are floral bract traits (Vanderhoeven *et al.* 2002; Blair and Hufbauer 2010; Vonica and Cantor 2011). Despite the low interspecific differentiation and high intraspecific polymorphism within this species complex (Hardy *et al.* 2000), capitula morphology differed significantly between individuals we genetically identified as putative parental taxa for three traits that characterized the colour and shape of the bract appendages **[see****Supporting Information—Fig. S1.1****]** as well as for the first principal component based on these traits. Moreover, holoploid genome size, which had previously been used as a taxonomically diagnostic trait to distinguish taxa with weak morphological differentiation (Mahelka *et al.* 2005; Ekrt *et al.* 2010), differed significantly between unadmixed *C*. cf. *jacea* and *C*. cf*. nigra* samples (Fig. 6A). However, we were not able to measure a key trait used previously to distinguish *C. nigra* from *C. jacea*—the absence of sterile ray florets (Vanderhoeven *et al.* 2002), and visual observations and vouchered specimens taken at the time of collection indicate that all populations in our New York samples possessed ray florets, suggesting they may in fact be products of advanced introgression rather than ‘pure’ *C. nigra* (L. Milbrath, pers. obs.). Therefore, the presence of ray-like florets at sites assigned to *C.* cf. *nigra* indicates that the parent species as described in various treatments is not present (or deviates from the current understanding), given that the lack of ray-like florets is considered one diagnostic trait for *C. nigra* (Vanderhoeven *et al.* 2002; Keil and Ochsmann 2006; Vonica and Cantor 2011).

Comparing individual assignments between the *K* = 2 and the most supported *K* = 3 Admixture runs (Fig. 2A), we found that two of the three identified clusters (red and blue clusters in Fig. 2A) comprised high numbers of relatively unadmixed individuals that were assigned to these clusters with >0.9 in both runs, whereas individuals assigned to the third cluster seemed to be of mixed ancestry (i.e. putative hybrids). These findings were supported by the genetic PCA analysis, which clearly separated the presumed parental taxa along PC1 with admixed individuals showing intermediate values (Fig. 3A–C). Most of the unadmixed individuals identified as genetically distinct in Admixture and PCA were also classified as parental types by NewHybrids (Fig. 2B), along with a preponderance of advanced generation (F2 and backcross) hybrids evident. This provides the best evidence to date for extensive introgression between *C. jacea* and *C. nigra*, but also suggests the persistence of relatively unadmixed genotypes that are morphologically similar to the parental taxa (at least *C. jacea*).

Unadmixed *C*. cf*. jacea* occurred exclusively in Vermont across seven populations, whereas populations with a predominantly unadmixed *C*. cf*. nigra* genetic background were more scarce and found in only one population in Vermont and three populations in New York (Figs 1 and 2). These results further reinforce the conclusion that the hybrid swarm of *C*. × *moncktonii* has retained some (though perhaps not all) genomic and morphological features of its presumed parental lineages, and that these can be confidently separated using a combination of genomic, morphological and cytological evidence.

### Genomic ancestry and geographic distribution of *Centaurea* × *moncktonii* populations

Previous studies have observed the presence of apparent *C*. × *moncktonii* hybrids in the Niagara region of New York (Eckel 2012), but we are unaware of any reports in the floristic literature on the origins of invasive populations in eastern North America—i.e. if they were introduced directly from Europe or are the result of a secondary introduction from the Pacific Northwest. Our study shows the presence of two types of *C*. × *moncktonii* hybrid populations in our study region, which is supported by both Admixture and genetic PCA analyses. One type of hybrid that is frequent in our New York samples corresponds to the ADMIXTURE lineage that emerges at *K* = 3 (orange ancestry in Figs 1 and 2A) and is assigned F2 or BC-jacea status in the NewHybrids analysis (Fig. 2B). This hybrid type also shared a higher amount of genomic ancestry with *C*. cf. *jacea* than *C*. cf. *nigra* based on the *K* = 2 results (Fig. 2A, upper). Both the closer relationship with *C*. cf*. jacea*, and also clear differentiation from the two presumed parental taxa are supported by the genetic PCA analysis (Fig. 3A, C and D). By contrast, the less abundant Vermont hybrid type is geographically restricted to just two of our sampled populations (SP and WR), and individual typically had ancestry assignments to all three clusters in the Admixture*K* = 3 analysis, with higher assignment to *C*. cf*. nigra* than to *C*. cf*. jacea* for *K* = 2 (Fig. 2A).

Whereas most of the Vermont hybrids are classified as advanced generation hybrids by NewHybrids based on both marker panels, assignment of the New York hybrids depended on the marker panel used. Based on the *C*. cf*. nigra* marker panel, New York hybrids were classified as predominantly F2’s or backcrosses to *C*. cf*. jacea* (Fig. 2B, upper), which corresponds well with the capitula morphology as well as with the Admixture results (*K* = 2). In contrast, hybrid class assignment based on the *C*. cf*. jacea* marker panel assigned most individuals as first or second generation backcrosses to *C*. cf*. jacea* (Fig. 2B, lower). The sensitivity of hybrid class assignments to the choice of marker panel likely reflects differences in the structure of linkage disequilibrium within each parental species genome, and the degree to which different maker sets are affected by shared ancestral polymorphism between species. Because the *C*. cf. *nigra* marker panel gave hybrid class assignments that more closely reflected the Admixture ancestry proportions (*K* = 2), and also gave results for the capitula morphometrics that fit with the degree of separation of the parental taxa, we suggest the analyses based on the *C*. cf*. nigra* marker panel are more robust. However, regardless of marker panel, both results point to advanced hybrid generations (either F2 or backcrosses) being commonplace.

The fact that we found almost exclusively advanced generation hybrids and no F1’s suggests that hybrid populations throughout our study region have persisted over multiple generations, and have diverged through either genetic drift or assortative mating. Our sampling in two regions of New York did not reveal any *C*. cf*. jacea* populations, although the hybrid individuals had higher genomic ancestry from this parental taxon than from *C*. cf*. nigra*. It is possible that *C*. cf*. jacea* has been completely introgressed in this region. Alternatively, New York hybrids may be descendants of a hybrid lineage formed in the more distant past (possibly prior to introduction) and now represents a stabilized hybrid gene pool. This latter scenario would seem to fit the genetic differentiation of this hybrid group along PC2 (Figs 2A and 3A, B and D), the very similar levels of admixed ancestry within and across these populations, and the trend towards smaller than expected genome sizes (Tamaki *et al.* 2017). Stable genomic composition in hybrids can be established within a few generations and thereafter be maintained over long periods, particularly if genomic rearrangements isolate the hybrid from mating with the parental taxa (Rieseberg 1997; Baack and Rieseberg 2007).

In contrast, all three identified hybrid populations in Vermont are located in the urban area of Burlington, which may have facilitated the more recent formation and persistence of hybrids through anthropogenic disturbance and/or gene flow through human infrastructure (Grabenstein and Taylor 2018). These populations may either have been formed through recent hybridization, as both parental taxa are still present in the region, or they may have been introduced via long distance dispersal, e.g. along traffic routes.

### Morphological and genome size variation in relation to genomic ancestry in *Centaurea* × *moncktonii* hybrids

Broadly speaking, strong concordance between genomic ancestry and taxonomic characteristics of genome size and capitula morphology was apparent in the hybrids, as exemplified by the intermediate mean values of various hybrid classes (Figs 5A, B and 6A) and the significant relationships of genetic with morphometric principal components as well as with genome size (Figs 5C, D and 6B). However, the hybrids encompassed considerable variation in genome size (Fig. 6), and capitula morphology exhibited transgressive segregation, exceeding the parental trait values, with the greatest variation observed in F2 and higher generation hybrids (Figs 4 and 5). The occurrence of transgressive phenotypes is a common phenomenon in advanced generation hybrid taxa after recombination (Rieseberg *et al.* 2003; Bell and Travis 2005) and may contribute to their success in novel environments (Baack and Rieseberg 2007; Hovick and Whitney 2014). Unequal crossing over or illegitimate recombination and other genomic rearrangements may also generate novel genetic architectures that increase trait variance in hybrids (Baack and Rieseberg 2007; Soltis *et al.* 2015). We observed advanced generation hybrids, particularly the F2+ hybrids, exhibited variable but smaller genome sizes on average relative to mid-parent expectations, with some individuals exhibiting the smallest genome sizes in the sample (Fig. 6). This could indicate progressive genomic downsizing in these hybrids, but whether or not smaller holoploid genome size leads to a higher invasive potential in *Centaurea* × *moncktonii*, as in other taxa (e.g. Pandit *et al.* 2014), awaits future experimental validation.

Interestingly, most hybrids exhibited a *C*. cf*. nigra*-like capitula morphology (Figs 4 and 5) although the majority of them (i.e. the New York hybrids) had a higher genomic ancestry from *C*. cf*. jacea* (Fig. 2A and B lower panel, see also **Supporting Information—Figs S2.1 and 2.2**). Mismatches between ancestry and morphology are not uncommon (Tamaki *et al.* 2017). Within the genus *Centaurea*, capitula traits have been studied in hybrids of diffuse and spotted knapweed (Blair *et al.* 2012), and suggested to be controlled by a small number of genes. Further, dominance relationships among the alleles determining the floral traits may skew the phenotype more towards one parental type (Fishman *et al.* 2002). These results emphasize the limitations of morphology for hybrid identification, and reinforce the utility of pairing ancestry data with genome size when discriminating parental and hybrid taxa, as genome size is unaffected by dominance.

### Conclusions and future perspectives

Invasive populations of *C. jacea*/*nigra* form a highly polymorphic species complex with previously unknown composition of genetic ancestry. By combining population genomics, genome size and morphometrics, our study revealed extensive introgression and a high abundance of advanced generation hybrids. However, in some locations relatively unadmixed populations of the putative parental taxa (*C*. cf*. jacea* and *C*. cf*. nigra*) persist. The potential for hybridization among co-invading reproductively compatible taxa is hypothesized to be a major mechanism for fuelling the establishment and spread of introduced plants (Hovick and Whitney 2014; Dlugosch *et al.* 2015). The extensive introgression we report here among these species of introduced *Centaurea* could increase their propensity for invasion if hybrids have higher short-term fitness (e.g. heterosis) or increased quantitative genetic variance (evolvability) compared to the parental types. This study provides a foundation for further work aimed at addressing the impact of admixture, introgression and changes in genome size on the fitness of invasive hybrids.

Increasing attention is being paid to the role of genomic and cytogenetic features, including genome size (Kubešová *et al.* 2010; Pandit *et al.* 2014; Suda *et al.* 2014; Schmidt *et al.* 2017) and genomic admixture following interspecific hybridization (Schierenbeck and Ellstrand 2009; Hovick and Whitney 2014) as potentially unifying traits promoting invasion success. The fact that species are transported globally with increasing frequency will lead to continued introduction of invasive hybrids to new regions as well as to the formation of novel hybrid taxa. Therefore, the hypothesized connection between hybridization, genome size variation and invasion success is likely to remain a critical issue for conservation of native species (Todesco *et al.* 2016) as well as fundamental to invasion and eco-evolutionary research (Hovick and Whitney 2014; Bock *et al.* 2015).

## Data

All data and code necessary to reproduce these results are publically available from https://github.com/SusanneLachmuth/Centaurea-moncktonii-AoB-Plants.

## Supporting Information

The following additional information is available in the online version of this article—

**Table S1.1.** Specimen voucher information for *Centaurea* samples analysed in this study. Vouchers have been archived at the Liberty Hyde Bailey Hortorium at Cornell University.

**Table S1.2.** Assignment criteria for *Centaurea* cf. *jacea* and *C.* cf. *nigra* and their hybrids using NEWHYBRIDS.

**Table S1.3.** List of measured and derived morphometric capitula traits diagnostic for the *Centaurea jacea/nigra* complex.

**Figure S1.1.** Boxplots of morphometric capitula traits (see also **Supporting Information—Table S1.2**) measured using standardized digital images of one or two capitula per individual collected in *Centaurea jacea/nigra* complex field populations in New York State and Vermont.

**Figure S1.2.** Comparison of the hybrid class assignments resulting from the NEWHYBRIDS analyses using each a set of 1000 selected single nucleotide polymorphism (SNP) loci that showed highest global *F*_ST_ and no linkage disequilibrium among either 27 *Centaurea* cf. *jacea* individuals or among 27 *C.* cf. *nigra* individuals.

**Figure S1.3.** Relationships of genetic ancestry and capitula morphology.

**Figure S1.4.** Relationships of genetic ancestry and genome size.

**Figure S2.1.** Genetic structure of 273 *Centaurea jacea/nigra* species complex individuals sampled in New York State and Vermont based on 10 348 single nucleotide polymorphisms (SNPs).

**Figure S2.2.** Principal component analysis based on three morphometric capitula traits.

**Figure S2.3.** Boxplots for variation in capitula morphology of the different hybrid classes.

**Figure S2.4.** Relationships of genetic ancestry and capitula morphology.

**Figure S2.5.** Relationships of genetic ancestry and genome size.

**Figure S2.6.** Relationships of capitula morphology and genome size.

plz055_suppl_Supplementary_Appendix_S1

plz055_suppl_Supplementary_Appendix_S2

## Sources of Funding

This work was supported by a United States Department of Agriculture Co-Operative agreement to J.M., S.R.K. and L.M. (58-1907-4-032), and by the German Research Foundation (DFG) through a Research Fellowship grant to S.L. (grant LA 3434/3-1).

## Contributions by the Authors

J.M., L.M., and S.R.K. planned and designed the study. J.M. and L.M. conducted the field sampling. J.S. performed the flow cytometry for genome size determination. J.M. and S.L. performed measurements of morphological traits. S.R.K. oversaw the G.B.S. library construction and sequencing. S.L. performed the bioinformatics and statistical analyses under guidance from S.R.K. S.L. and S.R.K. interpreted the results, with contributions from J.M. and L.M. S.L. and S.R.K. drafted the manuscript, and S.L., S.R.K., J.M. and L.M. contributed to the submitted version and revisions.

## Conflict of Interest

None declared.
