# Supplementary material for: Associations between genomic ancestry, genome size and capitula morphology in the invasive meadow knapweed hybrid complex (Centaurea × moncktonii) in eastern North America
Source: AoB Plants. 2019 Aug 23;11(5):plz055. doi: 10.1093/aobpla/plz055 (PMC6790064; doi:10.1093/aobpla/plz055)
Supplement: plz055_suppl_Supplementary_Appendix_S1 [file plz055_suppl_supplementary_appendix_s1.pdf]

Appendix S1

Contents:

Table S1.1: Specimen voucher information for *Centaurea* samples analyzed in this study. Vouchers have been archived at the Liberty Hyde Bailey Hortorium at Cornell University.

Table S1.2: Assignment criteria for *Centaurea* cf. *jacea* and *C.* cf. *nigra* and their hybrids using NEWHYBRIDS

Table S1.3: List of measured and derived morphometric capitula traits diagnostic for the *Centaurea jacea* / *nigra* complex

Figure S1.1: Boxplots of morphometric capitula traits (see also Table S1.2) measured using standardized digital images of one or two capitula per individual collected in *Centaurea jacea* / *nigra* complex field populations in New York State and Vermont

Figure S1.2: Comparison of the hybrid class assignments resulting from the NEWHYBRIDS analyses using each a set of 1000 selected SNP loci that showed highest global F_{ST} and no linkage disequilibrium among either 27 *C.* cf. *jacea* individuals or among 27 *C.* cf. *nigra* individuals

Figure S1.3: Relationships of genetic ancestry and capitula morphology

Figure S1.4: Relationships of genetic ancestry and genome size

Table S1.1: Specimen voucher information for *Centaurea jacea* s.l. samples analyzed in this study. Vouchers have been archived at the Liberty Hyde Bailey Hortorium at Cornell University.

Population ID	Accession	State	County	Collector
CB	117124	New York	Schoharie	J. Biazzo
CB	117132	New York	Schoharie	J. Biazzo
CB	117133	New York	Schoharie	J. Biazzo
CB	117134	New York	Schoharie	J. Biazzo
CB	117135	New York	Schoharie	J. Biazzo
CO	117128	New York	Schoharie	J. Biazzo
CO	117129	New York	Schoharie	J. Biazzo
CO	117130	New York	Schoharie	J. Biazzo
CO	117131	New York	Schoharie	J. Biazzo
CO	117430	New York	Schoharie	J. Biazzo
FL	117499	New York	Schuyler	J. Biazzo
FL	117500	New York	Schuyler	J. Biazzo
FL	117501	New York	Schuyler	J. Biazzo
FL	117502	New York	Schuyler	J. Biazzo
FL	117503	New York	Schuyler	J. Biazzo
FP	117367	New York	Montgomery	J. Biazzo
FP	117368	New York	Montgomery	J. Biazzo
FP	117369	New York	Montgomery	J. Biazzo
FP	117376	New York	Montgomery	J. Biazzo
FP	117377	New York	Montgomery	J. Biazzo
JF	116970	New York	Cortland	J. Biazzo
JF	117026	New York	Cortland	J. Biazzo
JF	117373	New York	Cortland	J. Biazzo
JF	117374	New York	Cortland	J. Biazzo
JF	117375	New York	Cortland	J. Biazzo
JV	116965	New York	Tompkins	J. Biazzo
JV	116966	New York	Tompkins	J. Biazzo
JV	116967	New York	Tompkins	J. Biazzo
JV	116968	New York	Tompkins	J. Biazzo
JV	116969	New York	Tompkins	J. Biazzo
LH	116964	New York	Schoharie	J. Biazzo
LH	117205	New York	Schoharie	J. Biazzo
LH	117206	New York	Schoharie	J. Biazzo
LH	117207	New York	Schoharie	J. Biazzo
LH	117208	New York	Schoharie	J. Biazzo
MC	117209	New York	Tompkins	J. Biazzo
MC	117210	New York	Tompkins	J. Biazzo
MC	117211	New York	Tompkins	J. Biazzo
MC	117370	New York	Tompkins	J. Biazzo
MC	117371	New York	Tompkins	J. Biazzo
MP	117121	New York	Tompkins	J. Biazzo

MP	117122	New York	Tompkins	J. Biazzo
MP	117123	New York	Tompkins	J. Biazzo
MP	117372	New York	Tompkins	J. Biazzo
WV	117425	New York	Tioga	J. Biazzo
WV	117426	New York	Tioga	J. Biazzo
WV	117427	New York	Tioga	J. Biazzo
WV	117428	New York	Tioga	J. Biazzo
WV	117429	New York	Tioga	J. Biazzo
CH	219916	Vermont	Chittenden	J. Molofsky
CH	219917	Vermont	Chittenden	J. Molofsky
CH	219918	Vermont	Chittenden	J. Molofsky
CH	219919	Vermont	Chittenden	J. Molofsky
DF	219938	Vermont	Addison	J. Molofsky
DF	219939	Vermont	Addison	J. Molofsky
FC	219937	Vermont	Addison	J. Molofsky
HV	219920	Vermont	Chittenden	J. Molofsky
HV	219921	Vermont	Chittenden	J. Molofsky
HV	219922	Vermont	Chittenden	J. Molofsky
PR	219913	Vermont	Chittenden	S. Keller
PR	219940	Vermont	Chittenden	S. Keller
RM	219914	Vermont	Chittenden	S. Keller
RM	219915	Vermont	Chittenden	S. Keller
SH	219923	Vermont	Grand Isle	J. Molofsky
SH	219924	Vermont	Grand Isle	J. Molofsky
SH	219925	Vermont	Grand Isle	J. Molofsky
SH	219926	Vermont	Grand Isle	J. Molofsky
SP	219927	Vermont	Chittenden	J. Molofsky
SP	219928	Vermont	Chittenden	J. Molofsky
SP	219929	Vermont	Chittenden	J. Molofsky
TH	219930	Vermont	Washington	J. Molofsky
TH	219931	Vermont	Washington	J. Molofsky
TH	219932	Vermont	Washington	J. Molofsky
TH	219933	Vermont	Washington	J. Molofsky
WR	219934	Vermont	Chittenden	J. Molofsky
WR	219935	Vermont	Chittenden	J. Molofsky
WR	219941	Vermont	Chittenden	J. Molofsky

Table S1.2: Assignment criteria for *Centaurea* cf. *jacea* and *C.* cf. *nigra* and their hybrids using NEWHYBRIDS (Anderson and Thompson, 2002) . Shown are the homozygous and heterozygous probabilities for each parental-type or hybrid category.

		**Genotype probability**			
**NEWHYBRIDS category**		***C.* cf. *jacea* (AA)**	**Heterozygote (AB)**	***C.* cf. *nigra* (BB)**	**NEWHYBRIDS category expanded**
C. cf. *jacea*	Parental taxon	0	0	1	*C*. cf. *jacea*
C. cf. *nigra*	Parental taxon	1	0	0	*C*. cf. *nigra*
F1	First generation hybrid	0	1	0	(*C*. cf. *jacea* x *C*. cf. *nigra*)
BC1 jacea	First generation backcross	0	0.5	0.5	*C*. cf. *jacea* x (*C*. cf. *jacea* x *C*. cf. *nigra*)
BC1 nigra	First generation backcross	0.5	0.5	0	*C*. cf. *nigra* x (*C*. cf. *jacea* x *C*. cf. *nigra*)
F2*	Second generation hybrid	0.25	0.5	0.25	(*C*. cf. *jacea* x *C*. cf. *nigra*) x (*C*. cf. *jacea* x *C*. cf. *nigra*
BC2 jacea*	Second generation backcross	0	0.25	0.75	*C*. cf. *jacea* x (*C*. cf. *jacea* x (*C*. cf. *jacea* x *C*. cf. *nigra*))
BC2 nigra*	Second generation backcross	0.75	0.25	0	*C*. cf. *nigra* x (*C*. cf. *nigra* x (*C*. cf. *jace*a x *C*. cf. *nigra*))

*Denotes recombinant hybrid categories that cannot be distinguished from more advanced generation recombinants (e.g., F3, BC3, F4, etc).

Table S1.3: List of measured and derived morphometric capitula traits that are a subset of recognized traits diagnostic for the *Centaurea jacea / nigra* complex. We based our trait selection on a subset of characteristics listed in the *Flora of North America* (Keil and Ochsmann, 2006) or that have been diagnostic in previous morphometric analyses on European samples of the species complex (Vanderhoeven et al., 2002), and that could be reliably measured from standardized digital images.

**Derived trait**	**Measured traits**	**Morphology of the potential parental taxa according to literature reco**	
		Centaurea jacea	***Centaurea nigra***
Number of bract rows	Nr. of bract rows	Capitulum about as long as wide[1]	Capitulum as long as wide[1]
Pectinate bract rows (%)	Nr. of bract rows<br>Nr. of pectinate bract rows	Bracts entire to coarsely dentate, membraneous[1]	Bracts pectinate[1]
Bract color[2]	Bract color (1, black; 2, intermediate; 3, light brown)[2]	Light brown[1]	Dark brown to black[1]
Capitum width / length ratio	Width of capitulum<br>Length of capitulum	Capitulum about as long as wide[1]	Capitulum as long as wide[1]
Appendage center width / length ratio	Width of central undivided part of bract appendage[2]<br>Length of central undivided part of bract appendage[2]	Bract appendages roundish[1]	Undivided bract appendage cente egg-shaped roundish[3]
Relative bract appendage center length	Width of central undivided part of bract appendage<br>Width of bract	Trait reflects 'length of apical bract tooth'[2]	

1) Keil and Ochsmann, 2006, 2)Vanderhoeven et al., 2002; 3) Jäger, 2011

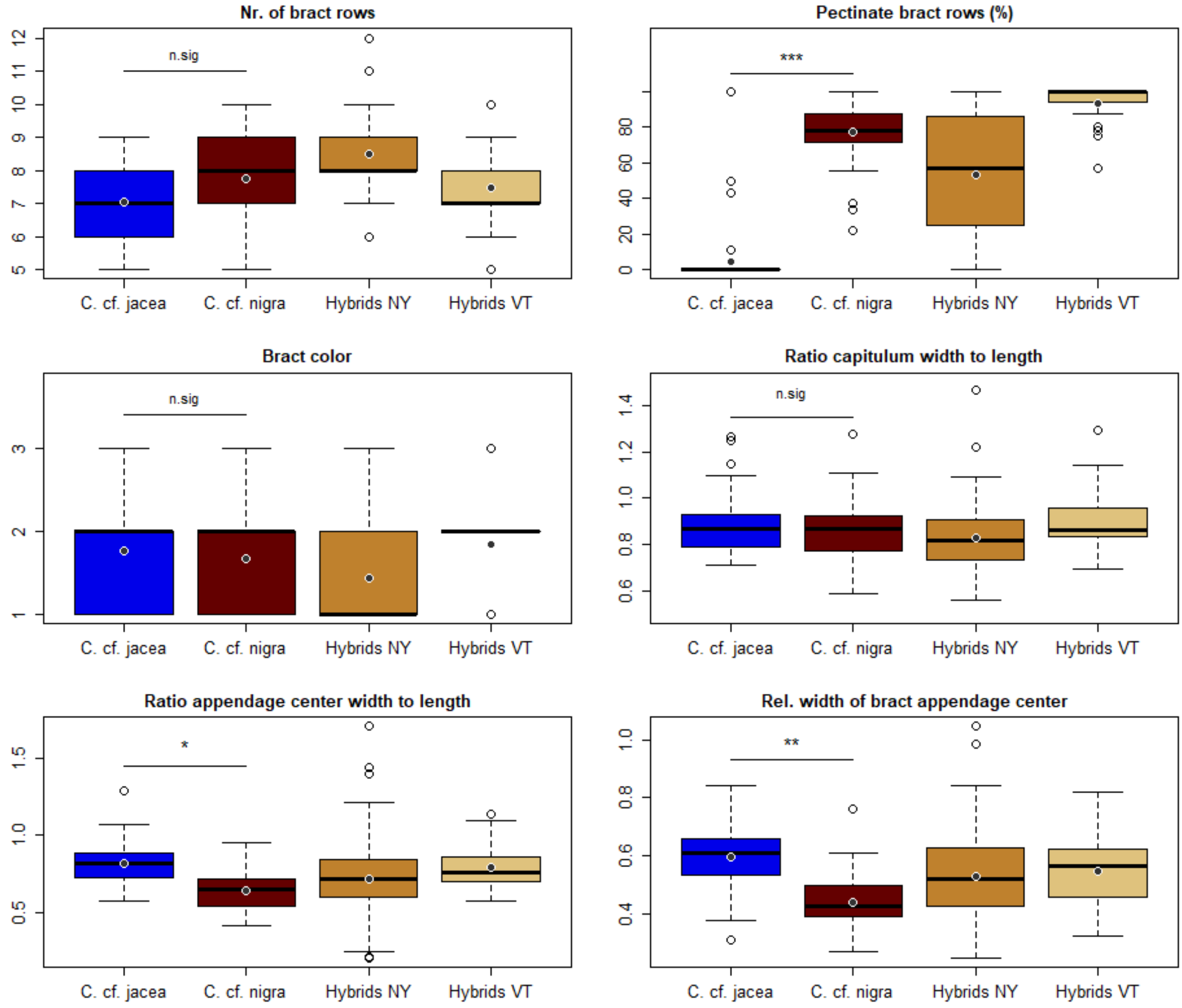

Figure S1.1: Boxplots of morphometric capitula traits (see also Table S1.2) measured using standardized digital images of one or two capitula per individual collected in *Centaurea jacea / nigra* complex field populations in New York State (NY) and Vermont (VT) (see Fig. 1, Table 1). Samples were grouped into categories based on ADMIXTURE analysis representing *C.* cf. *jacea and C.* cf. *nigra* (unadmixed samples with ancestry probability > 0.9) as well as hybrids from NY and Vermont VT. Bract color is a qualitative character with three levels (1, black; 2, intermediate; 3, light brown; according to (Vanderhoeven et al., 2002). Results of mixed model comparisons of the *C.* cf. *jacea* and *C.* cf. *nigra* mean values are given: n.sig.: p >0.05, *: p < 0.05, **: p < 0.01, ***: p < 0.001.

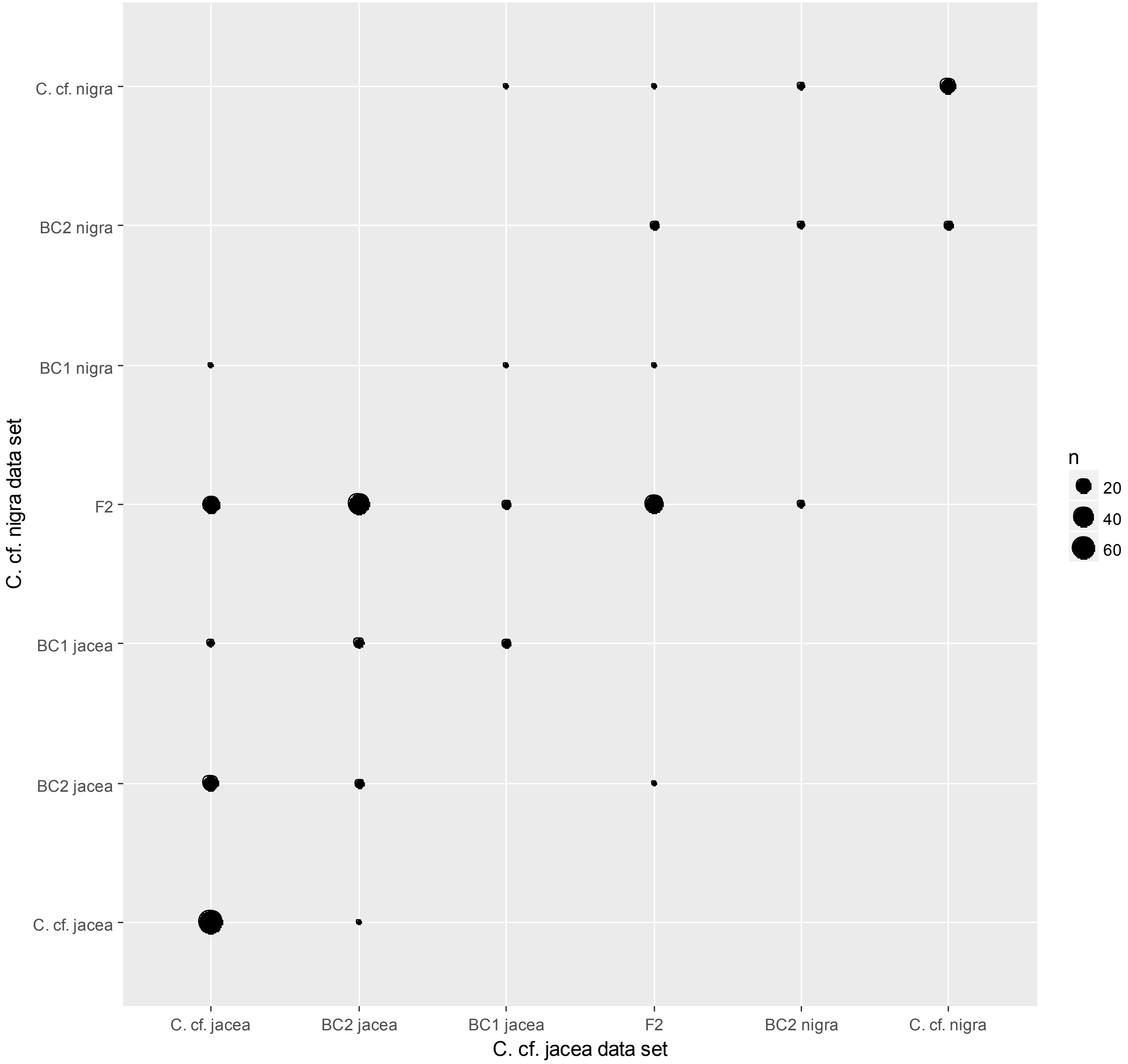

Figure S1.2: Comparison of hybrid class assignments resulting from the NEWHYBRIDS analyses (Anderson and Thompson, 2002) based on different marker panel data sets. Each a set consisted of 1000 SNP loci that showed the highest global F_{ST} and no linkage disequilibrium among either 27 *C.* cf. *jacea* individuals (*C.* cf. *jacea* data set; see also Fig. 2b, lower panel) or among 27 *C.* cf. *nigra* individuals (*C.* cf. *nigra* data set; see also Fig. 2b, upper panel). Size of the dots represents the number (n) of samples that were assigned to each combination of hybrid classes between the two data sets. C. cf. jacea / C. cf. nigra, parental taxa; BC1 jacea / BC1 nigra, first generation backcrosses towards parental taxa; BC2 jacea / BC2 nigra, second or higher generation backcrosses towards parental taxa; F2, second or higher hybrid inter-cross generations.

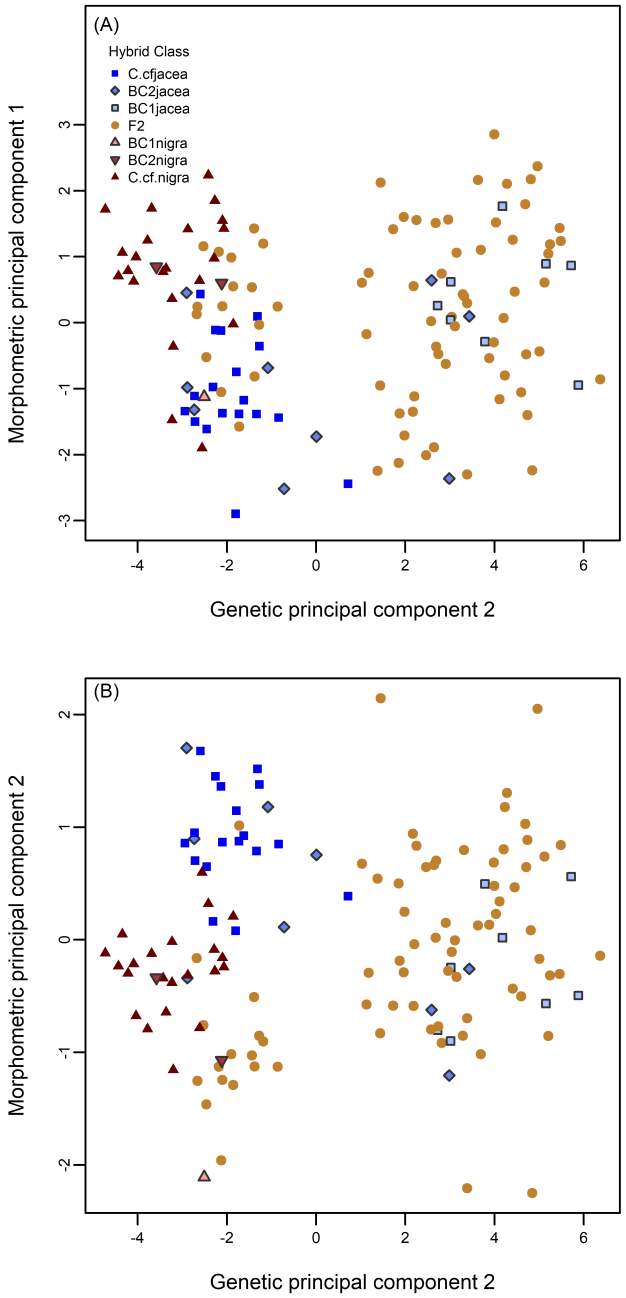

Figure S1.3: Relationships of genetic ancestry and capitula morphology. Mean individual scores for principal component 1 (A) and 2 (B) from the morphometric PCA presented in Fig. 4 as a function of individual scores for principal component 2 from the genetic PCA presented in Fig. 3. The colored labels correspond to hybrid classes derived from the NEWHYBRIDS analysis using the *C.* cf. *nigra* marker panel (see Fig. 2B, upper panel). These two relationships were not significant in mixed model analyses.

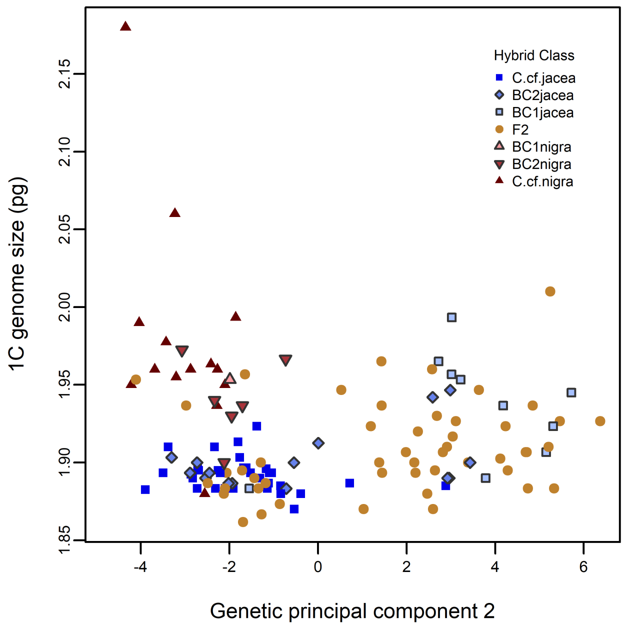

Figure S1.4: Relationships of genetic ancestry and genome size. Mean offspring genome size (1C value) as a function of individual scores for principal component 2 from the genetic PCA presented in Fig. 3. Each point represents an individual sample in the genomic analyses. Colored labels correspond to hybrid classes derived from the NEWHYBRIDS analysis based on the 27 *C.* cf. *nigra* marker panel (see Fig. 2B, upper panel). This relationship was not significant in mixed model analyses.

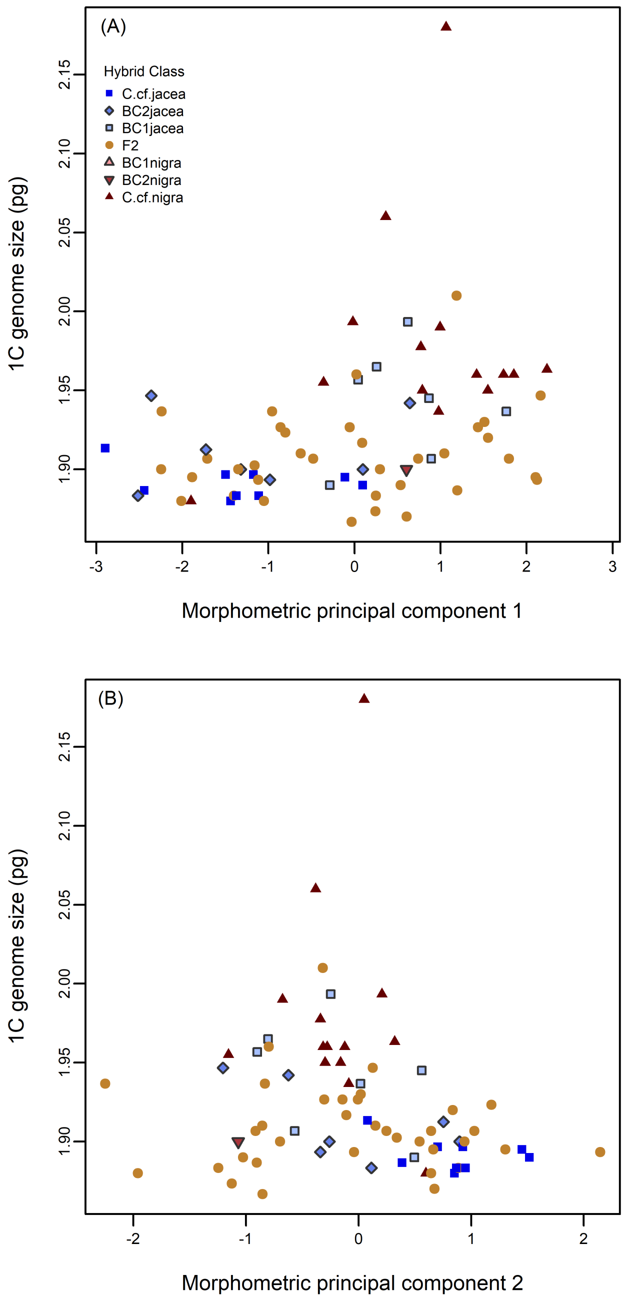

Figure S1.5: Relationships of capitula morphology and genome size. Mean offspring genome size (1C value) was tested as a function of mean individual scores for principal component 1 (A) and 2 (B) from the morphometric PCA presented in Fig. 4. Each point represents an individual as included in the morphometric analyses. Colored labels correspond to hybrid classes derived from the NEWHYBRIDS analysis using the *C.* cf. *nigra* marker panel (see Fig. 2B, upper panel). These two relationships were not significant in mixed model analyses.

References

ALEXANDER, D.H., J. NOVEMBRE, and K. LANGE. 2009. Fast model-based estimation of ancestry in unrelated individuals. *Genome Research* 19: 1655–1664.

ANDERSON, E.C., and E.A. THOMPSON. 2002. A Model-Based Method for Identifying Species Hybrids Using Multilocus Genetic Data. *Genetics* 160: 1217–1229.

JÄGER, E.J. ed. . 2011. Exkursionsflora von Deutschland, begründet von W. Rothmaler, Gefäßpflanzen: Grundband. Spektrum Akademischer Verlag, Heidelberg.

KEIL, D.J., and J. OCHSMANN. 2006. Centaurea Linnaeus. *In* Flora of North America. Vol. 19, 20 and 21, pp. 52, 57, 58, 67, 83, 84, 96, 171, 172, 176, 177, Available at: http://www.efloras.org/florataxon.aspx?flora_id=1&taxon_id=106012 (accessed 18.12.2017).

VANDERHOEVEN, S., O. HARDY, X. VEKEMANS, C. LEFÈBVRE, M. DE LOOSE, J. LAMBINON, and P. MEERTS. 2002. A Morphometric Study of Populations of the Centaurea jacea Complex (Asteraceae) in Belgium. *Plant Biology* 4: 403–412.
