# Supplementary material for: Associations between genomic ancestry, genome size and capitula morphology in the invasive meadow knapweed hybrid complex (Centaurea × moncktonii) in eastern North America
Source: AoB Plants. 2019 Aug 23;11(5):plz055. doi: 10.1093/aobpla/plz055 (PMC6790064; doi:10.1093/aobpla/plz055)
Supplement: plz055_suppl_Supplementary_Appendix_S2 [file plz055_suppl_supplementary_appendix_s2.pdf]

Appendix S2

Figures with NEWHYBRIDS hybrid class assignments based on the *Centaurea* cf. *jacea* marker panel

Contents:

Figure S2.1: Genetic structure of 273 *Centaurea jacea / nigra* species complex individuals sampled in New York State and Vermont based on 10,348 SNPs.

Figure S2.2: Principal component analysis based on three morphometric capitula traits

Figure S2.3: Boxplots for variation in capitula morphology of the different hybrid classes

Figure S2.4: Relationships of genetic ancestry and capitula morphology

Figure S2.5: Relationships of genetic ancestry and genome size

Figure S2.6: Relationships of capitula morphology and genome size

Appendix S2

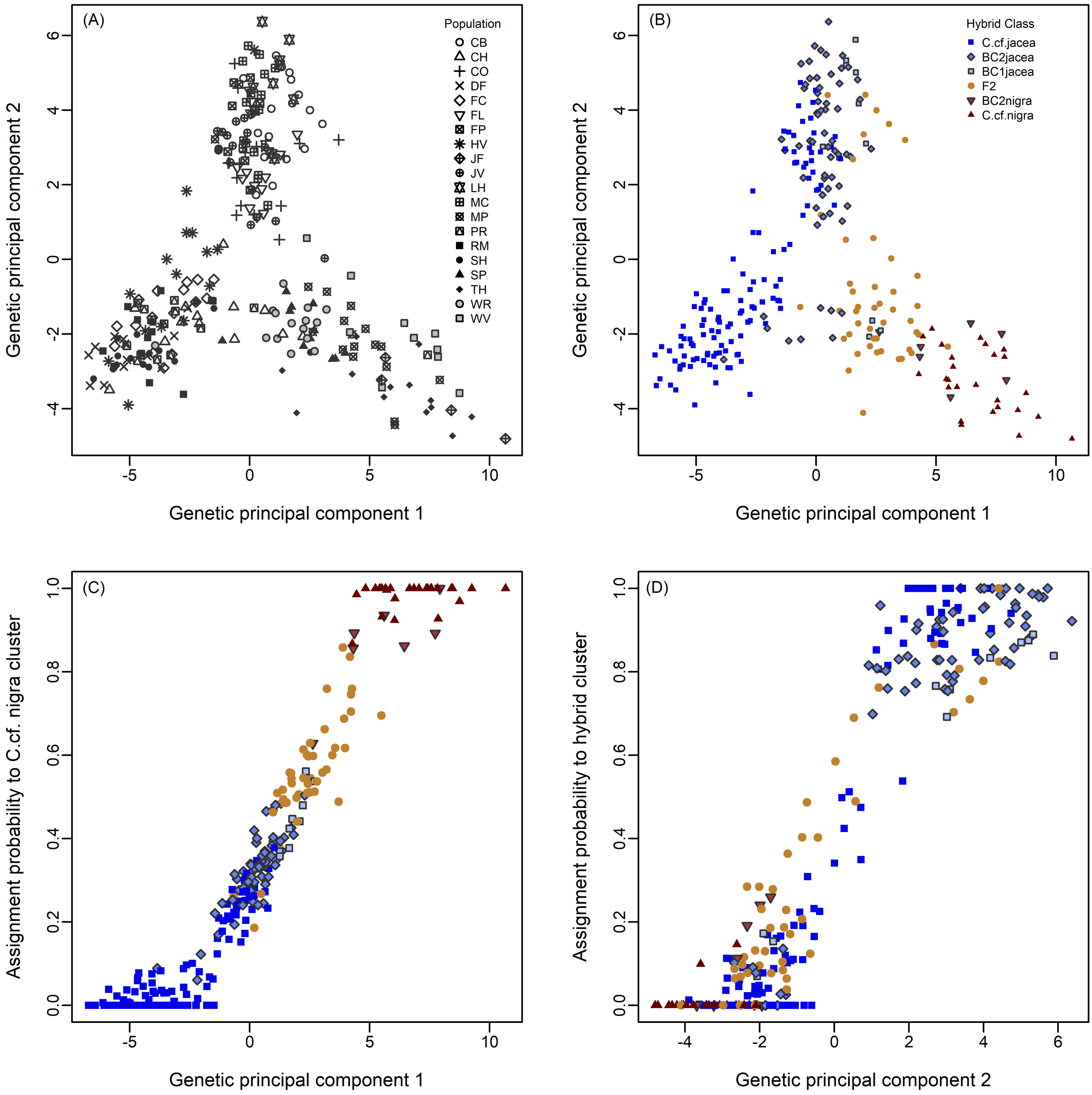

Figure S2.1: Genetic structure of 273 *Centaurea jacea / nigra* species complex individuals sampled in New York State and Vermont based on 10,348 SNPs. (A,B) Principal component analysis labeled by population as listed in Table 1 (A) and NewHybrids (Anderson and Thompson, 2002) hybrid classes (B). (C) Relationship between the assignment probability to the *C.* cf. *nigra* (red) cluster as estimated by the Admixture analysis (Alexander et al., 2009) for K=2 (see Fig. 2A, upper panel) and the scores of principal component 1 (see A, B). (D) Relationship between the assignment probability to the *C.* ×*moncktonii* hybrid cluster (orange) as estimated by the Admixture analysis for K=3 (see Fig. 2A, lower panel) and the scores of principal component 2 (see A, B). The hybrid classes represented by colored labels were derived from the NewHybrids analysis (Anderson and Thompson, 2002) using a set of 1000 selected SNP loci that showed highest global F_{ST} and no linkage disequilibrium among 27 *C.* cf. *jacea* individuals (see Fig. 2B, lower panel): C. cf. jacea / C. cf. nigra, parental taxa; BC1 jacea / BC1 nigra, first generation backcrosses towards parental taxa; BC2 jacea / BC2 nigra, second or higher generation backcrosses towards parental taxa; F2, second or higher hybrid inter-cross generations.

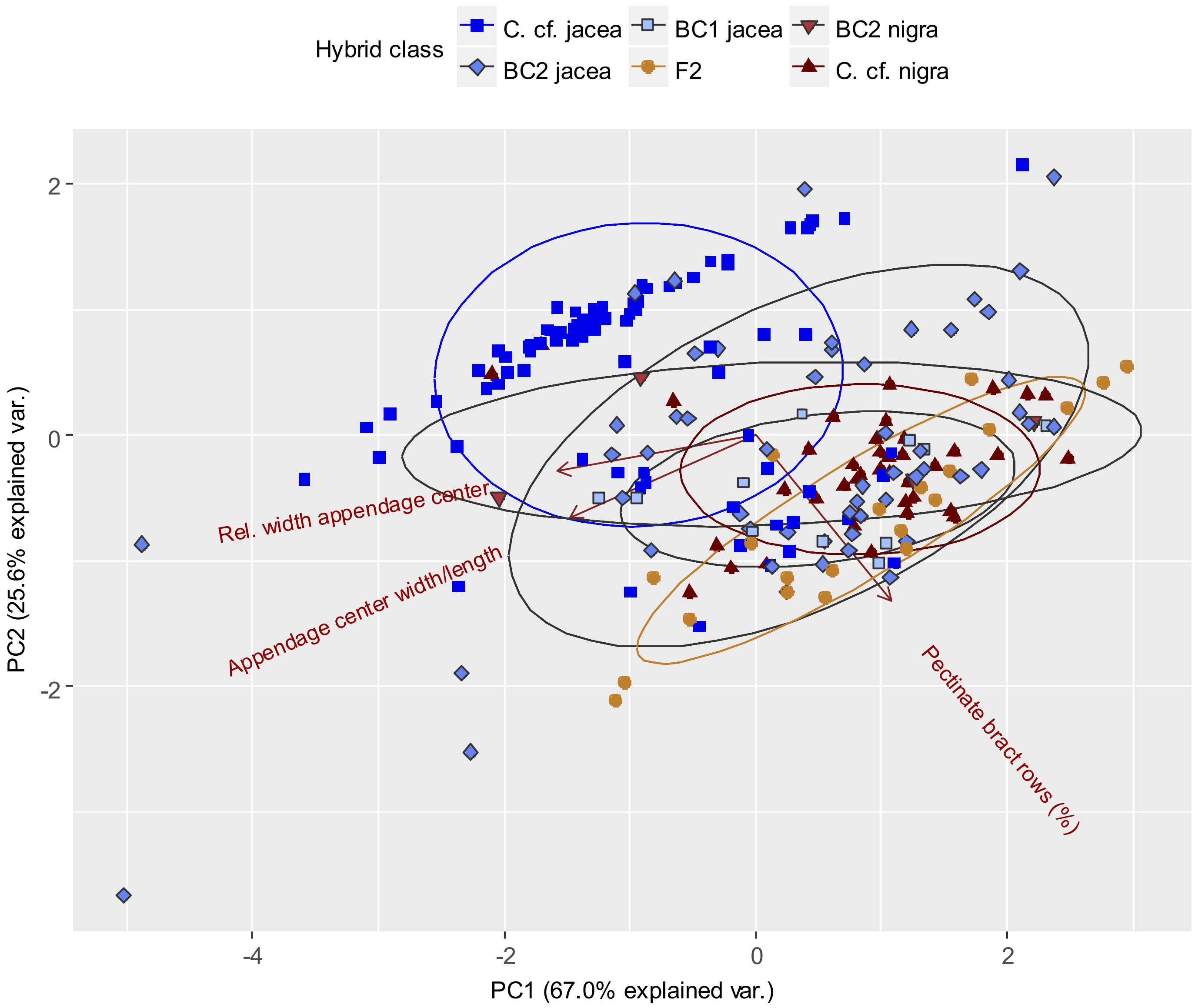

Figure S2.2: Principal component analysis based on three morphometric capitula traits (see Table S1.2 and Fig. S1.1) derived from a subset of diagnostic characteristics from previous morphometric analyses on European samples of the *Centaurea jacea / nigra* complex (Hardy et al., 2000; Vanderhoeven et al., 2002). The traits were measured for 133 individuals using standardized digital images of one or two field collected capitula per individual and showed significant variation (see Fig. S1.1) between the *C.* cf. *jacea* and *C.* cf. *nigra* individuals as identified by the ADMIXTURE analysis (Alexander et al., 2009) for K=2 presented in Fig. 2A. Each dot represents a capitulum. The colored labels correspond to hybrid classes derived from the NEWHYBRIDS analysis using the *C.* cf. *jacea* marker panel (see Fig. 2B, lower panel).

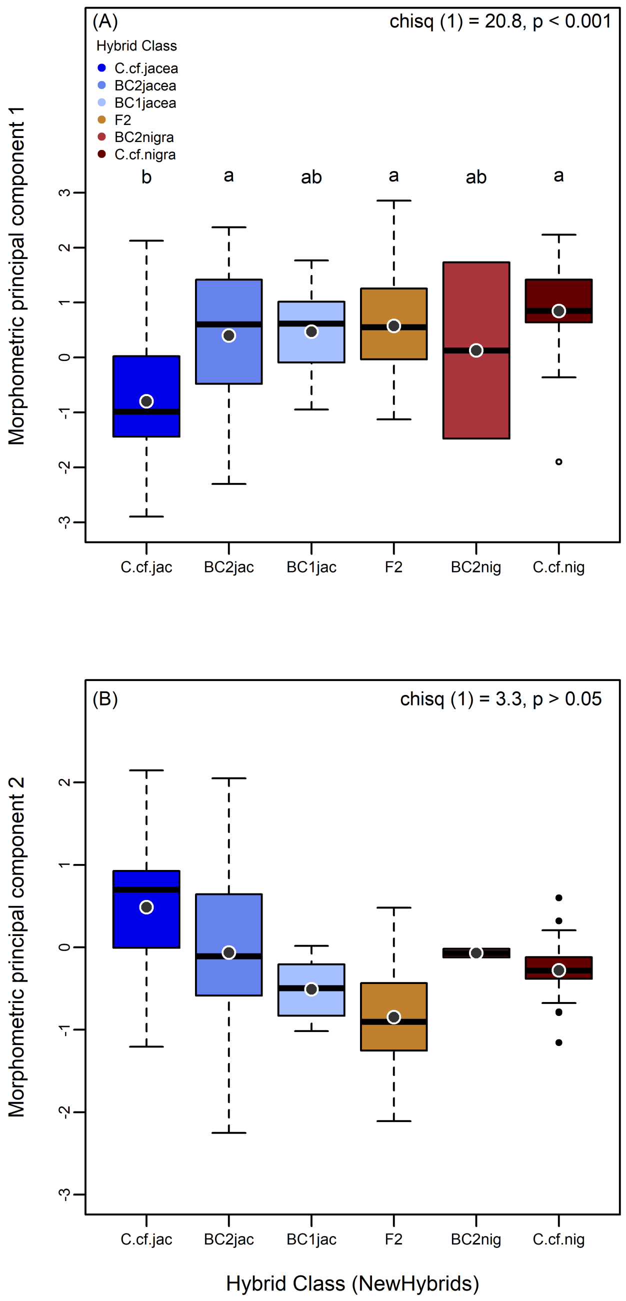

Figure S2.3: Boxplots for variation in capitula morphology for the different hybrid classes. Mean individual scores for principal component 1 (A) and 2 (B) from the morphometric PCA presented in Fig. S2.2 as a function of hybrid class. Hybrid classes were derived from the NEWHYBRIDS analysis using the *C.* cf. *jacea* marker panel (see Fig. 2B, lower panel). Significance of the hybrid class main effect was tested using likelihood ratio test for one-way ANOVAs performed with mixed models including a random effect of population. Hybrid classes that do not share a lower-case letter differ significantly ($p < 0.05$) according to Tukey's post-hoc tests.

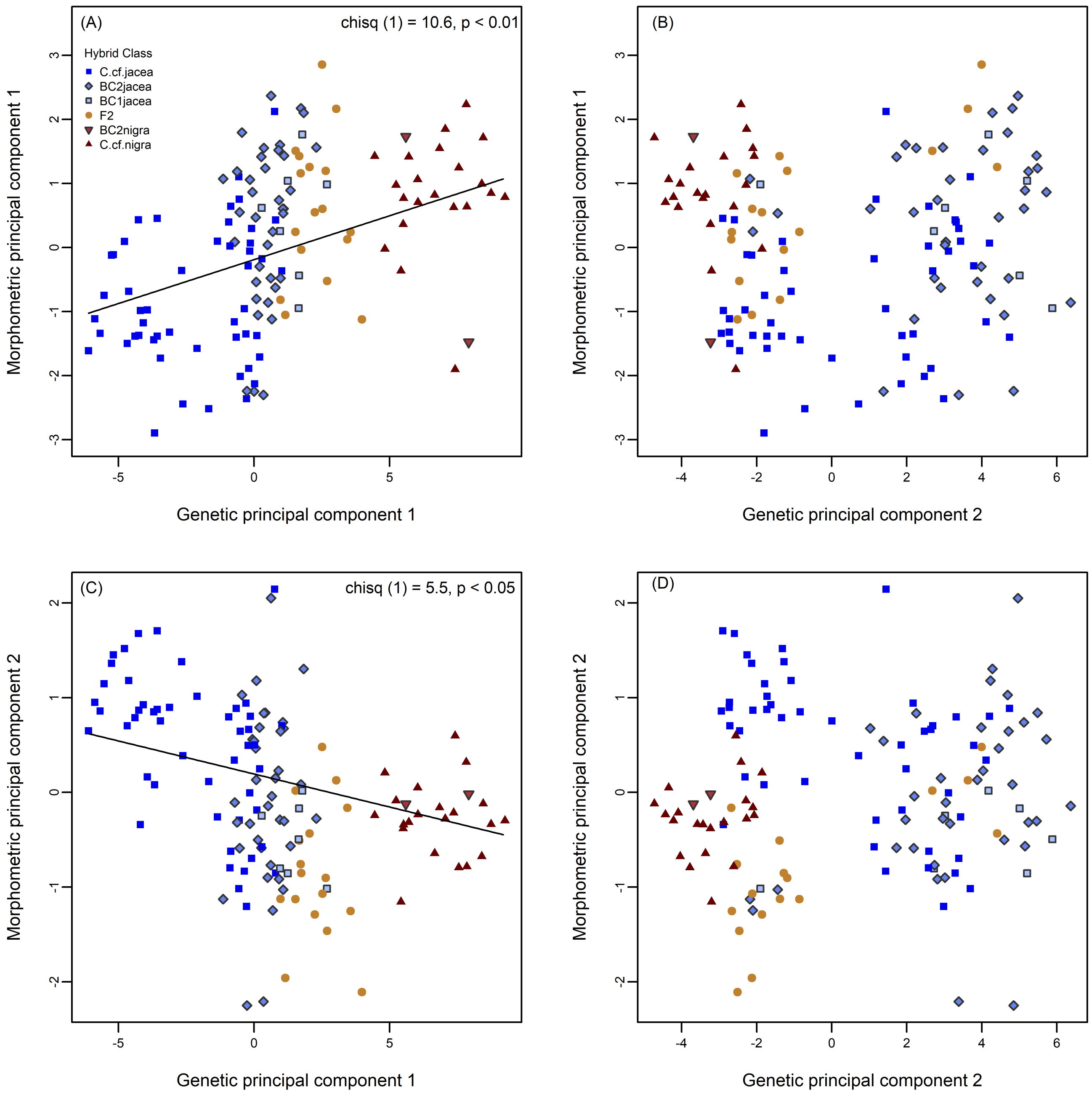

Figure S2.4: Relationships of genetic ancestry and capitula morphology. Mean individual scores for principal component 1 (A, B) and 2 (C, D) from the morphometric PCA presented in Fig. S2.2 as a function of individual scores for principal component 1 (A, C) and 2 (B, D) from the genetic PCA presented in Fig. S2.1. Each dot represents an individual. The colored labels correspond to hybrid classes derived from the NEWHYBRIDS analysis using the *C.* cf. *jacea* marker panel (see Fig. 2B, lower panel). Lines represent model predictions of single predictor mixed models including a random effect of population (only for significant effects). Significance was tested using likelihood ratio tests.

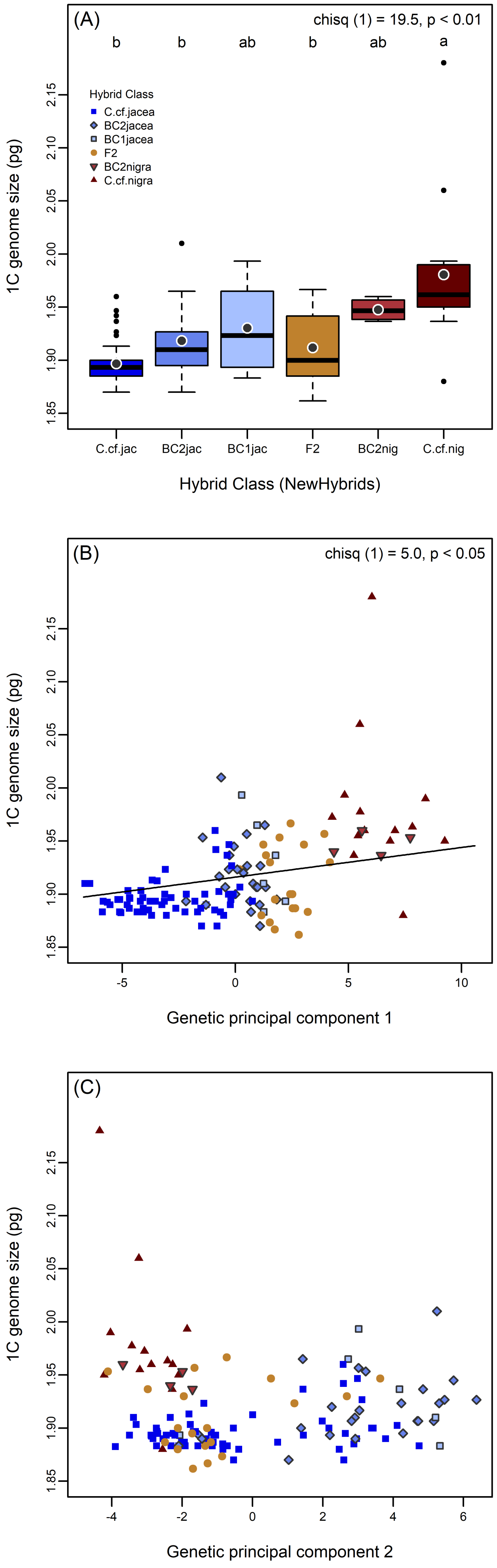

Figure S2.5: Relationships of genetic ancestry and genome size. Mean offspring genome size (1C value) was tested as a function of NEWHYBRIDS hybrid class (A) as well as individual scores for principal component 1 (B) and 2 (C) from the genetic PCA presented in Fig. S2.1. Each point represents an individual. The colored labels correspond to hybrid classes derived from the NEWHYBRIDS analysis using the *C.* cf. *jacea* marker panel (see Fig. 2B, lower panel). Significance of main effects was tested using likelihood ratio tests in single-predictor mixed models including a random effect of population. Lines represent model predictions. Hybrid classes that do not share a lower-case letter differ significantly ($p < 0.05$) according to Tukey's post-hoc tests.

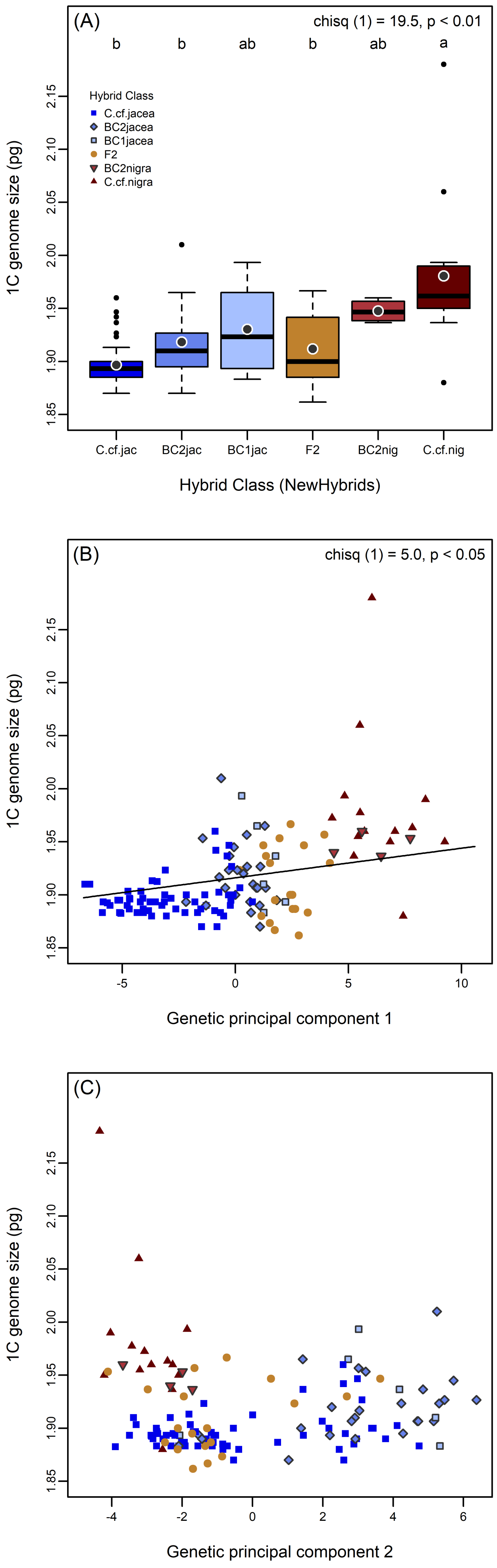

Figure S2.6: Relationships of capitula morphology and genome size. Mean offspring genome size (1C value) was tested as a function of mean individual scores for principal component 1 (A) and 2 (B) from the morphometric PCA presented in Fig. S2.2. Each point represents an individual. Colored labels correspond to hybrid classes derived from the NEWHYBRIDS analysis using the *C.* cf. *jacea* marker panel (see Fig. 2B, lower panel). These two relationships were not significant in mixed model analyses including a random effect of population.

Appendix S2

References

ALEXANDER, D.H., J. NOVEMBRE, and K. LANGE. 2009. Fast model-based estimation of ancestry in unrelated individuals. *Genome Research* 19: 1655–1664.

ANDERSON, E.C., and E.A. THOMPSON. 2002. A Model-Based Method for Identifying Species Hybrids Using Multilocus Genetic Data. *Genetics* 160: 1217–1229.

HARDY, O.J., S. VANDERHOEVEN, M. DE LOOSE, and P. MEERTS. 2000. Ecological, morphological and allozymic differentiation between diploid and tetraploid knapweeds (Centaurea jacea) from a contact zone in the Belgian Ardennes. *New Phytologist* 146: 281–290.

VANDERHOEVEN, S., O. HARDY, X. VEKEMANS, C. LEFÈBVRE, M. DE LOOSE, J. LAMBINON, and P. MEERTS. 2002. A Morphometric Study of Populations of the Centaurea jacea Complex (Asteraceae) in Belgium. *Plant Biology* 4: 403–412.
